# Use of ammonium salts or binary mixtures derived from amino acids, glycine betaine, choline and indole-3-butyric acid as plant regulators†

**DOI:** 10.1039/d0ra09136g

**Published:** 2020-11-26

**Authors:** Damian Krystian Kaczmarek, Anna Parus, Marek Łożyński, Juliusz Pernak

**Affiliations:** Faculty of Chemical Technology, Poznan University of Technology Berdychowo 4 Poznan 60-965 Poland juliusz.pernak@put.poznan.pl

## Abstract

A simple, efficient, and environmentally friendly synthesis method for bioproducts based on indole-3-butyric acid and amino acids, glycine betaine or choline has been developed. Spectral analysis and molecular calculations were used to determine whether the products were ammonium salts or binary mixtures. Moreover, it was observed that the ammonium salts degraded more rapidly than the binary mixtures when exposed to light. The structures of the products significantly impacted their thermal stability and phase transitions. Biological studies clearly showed that the synthesized products were more effective than a reference commercial preparation as a rooting agent and have significant potential as new biologically active agents with low environmental impact.

## Abstract 2

Natural origin ammonium salts or binary mixtures including indole-3-butyric acid as novel plant growth regulators.

## Graphical abstract

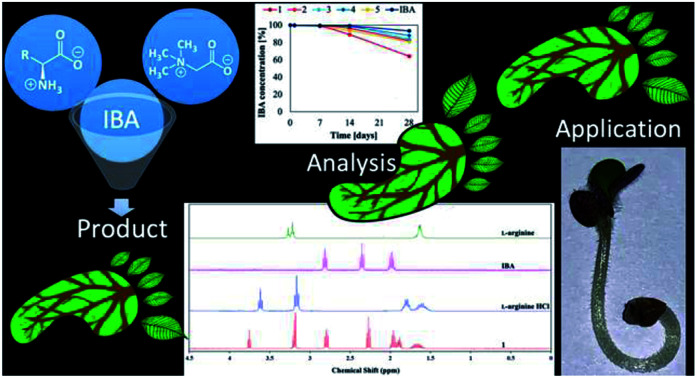

## Introduction

Green chemistry is the production of novel compounds with reduced environmental impact to eliminate or limit pollutant generation. Energy consumption and production costs must be considered in the design of novel ‘green’ compounds.^1^ Ammonium salts (including ionic liquids, ILs)^2–6^ or binary mixtures (including eutectic mixtures)^7–10^ are perfectly compatible with this trend. However, not every IL or binary mixture conforms to green chemistry guidelines. Therefore, suitable ions or components must be selected to design appropriate structures that reduces the environmental impact of novel products. Amino acids are ideal substrates for the synthesis of ammonium salts or binary mixtures: these bifunctional organic compounds exist in anionic, cationic or zwitterionic forms depending on the pH.^2–6^ Amino acids and their ionic forms offer advantages of a natural origin, nontoxicity and biocompatibility. Thus, amino acids are used as dietary supplements, additives in animal feed, and plant nutrition, among many other applications.^11–14^ However, choline^15,16^ and glycine betaine^17,18^ are the most frequently used natural compounds because of price considerations. Being natural in origin, choline and glycine betaine are environmentally friendly. These compounds are used in many areas of chemistry in the form of ammonium salts or mixtures.^19,20^

The current pandemic has largely eclipsed other problems worldwide.^21^ One such problem is population growth and access to food in some regions of the planet. Various types of plant protection products have been used to increase agricultural production for many years.^22^ However, not all these substances have proved to be safe many years after application, and safer forms are needed. Natural plant growth regulators are potential alternatives.^23,24^

Indole-3-butyric acid (IBA) is a naturally derived auxin produced by plants and contributes to root elongation or leaf epinasty. IBA is generally nontoxic to plants and living organisms; however, excess IBA is detrimental to plant development. IBA has a low water solubility and is therefore difficult to use in agriculture. Nevertheless, IBA is widely used as a rooting agent and a component of plant growth stimulators. By contrast, scientific studies on IBA and ammonium salts or eutectics have mainly focused on the extraction of auxins and other compounds of natural origin from plants.^25^ To the best of our knowledge, the only study on using IBA (in the form of ammonium salts) as a plant growth regulator was published in 2020.^26^

The main aim of this study was to develop a synthesis method for an amino acid (l-arginine, l-histidine, l-proline), glycine betaine or choline and indole-3-butyric acid. It was determined whether the selected substrates occurred as protic ammonium salts or binary mixtures. Spectroscopic techniques and molecular calculations were used to establish the structures of and the interactions between the molecules. The influence of light and temperature on the decomposition of the synthesized products was determined. Finally, biological tests were conducted to assess the effect of the tested substances on germination and plant development.

## Results and discussion

The novel products were synthesized using substrates such as l-arginine, l-histidine, l-proline, glycine betaine, choline and IBA. The synthesis process consisted of reacting IBA with another compound in a 1 : 1 molar ratio in a mixture of solvents (water : ethanol at 10 : 1 v/v) at 40 °C for 24 hours in the dark solvents were removed, and the products were protected from exposure to light and water (see the ESI for a detailed description of the synthesis†). The water content of all the products was measured to be below 500 ppm. The developed synthesis method yielded five reaction products 1–5: 1, 2 and 3 were white solids, and 4 was obtained as a wax. The synthesized ionic liquid (5) was obtained following a methodology described in the literature^26^ and used together with IBA as reference substances (Table 1).

**Table 1:** Products synthesized using IBA

No.	Cation/component	pH of aqueous solutions (1%)	*T*_m_ a [°C]	*T*_c_ b [°C]	*T*_g_ c [°C]	*T*_0.05_ d [°C]
1	l-Arginine	4.94	181	—	—	351
2	l-Histidine	5.61	110	93	—	348
3	l-Proline	6.02	112	—	13	290
4	Betaine	7.23	—	—	2	218
5^e^	Choline	7.03	—	—	−18.0	207
IBA	—	3.54	120	—	—	200

a *T*_c_ – crystallization temperature.

b *T*_m_ – melting point.

c *T*_g_ – glass transition temperature.

d *T*_0.05_ – decomposition temperature of 5% sample.

e Data from literature.^26^

The measured pH values of the aqueous solutions given in Table 1 led us to speculate that ammonium salts 1 and 2 could be produced by reacting IBA with l-arginine and l-histidine, where the amino acids were in cationic form. It has been reported that l-proline exists as a zwitterion at a pH of approx. 6;^27,28^ therefore, the measured pH of product 3 (6.02) indicated that the obtained product should be a binary mixture. A neutral pH was measured for aqueous solutions of product 4 and ionic liquid 5. In the following sections, we prove the hypothesis that products 1 and 2 are ammonium salts and products 3 and 4 are binary mixtures.

Molecular calculations and an analysis of UV-Vis and ^1^H and ^13^C NMR spectra were performed to determine the type of interactions between the product components and to confirm the structure of products 1–4. Only the NMR and UV-Vis spectra were analysed for ionic liquid 5, which has already been characterized in the literature. The UV-Vis, ^1^H and ^13^C NMR spectra are presented in Fig. S1–S14 of the ESI.†

**Fig. 1:**
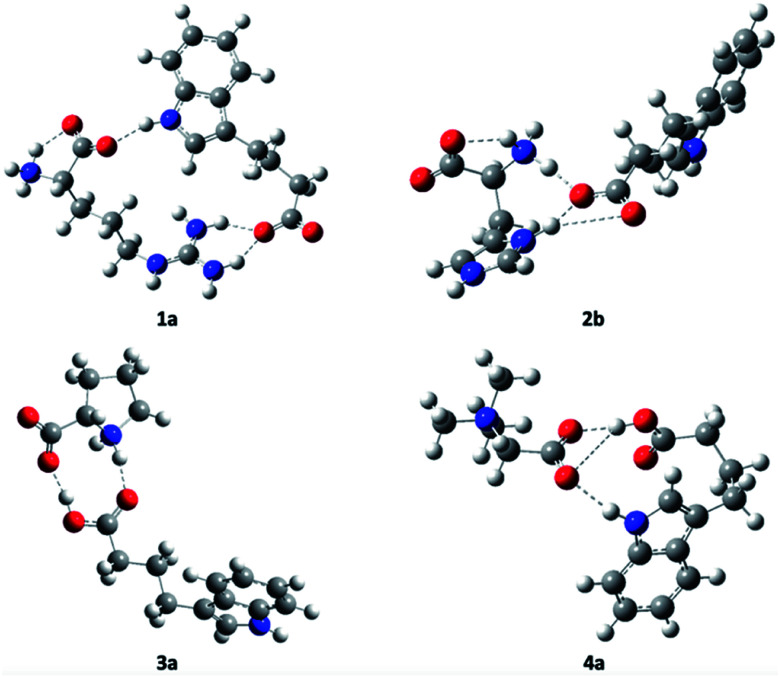
Optimized structures of products 1, 2, 3, and 4.

All the components of the complexes of l-arginine, l-histidine, and glycine-betaine (except l-proline) with IBA include at least two functional groups active in hydrogen bonding. By contrast, l-proline is a cyclic and mono-functional amino acid that forms a zwitterionic secondary amine. That is, these complexes exhibit a variety of hydrogen bonding (HB) patterns. Different intermolecular associations may occur depending on whether the solutions of the IBA derivative are concentrated or dilute. It is reasonable to consider cyclic dimer structures for 1a, 2a, 3a, and 4b in dilute aqueous solutions (Fig. 1). Moreover, one cannot exclude the high affinity of the cationic ammonium, guanidinium, and imidazolium systems towards the indole group to form complexes such as products 1_2_, 2_2_, or 4_2_, where a single amino acid molecule forms a linear complex with two carboxylate molecules (all the proposed structures are presented in Fig. 1 and S15 in the ESI†). The simulations were performed to identify zwitterion-anion associations and rank the association strengths of potential ion pairs (the detailed molecular computation method is presented in the ESI†). Undoubtedly, these associations are driven by attractive electrostatic forces between ion pairs and, if water does not intervene, hydrogen bonds. Consistent with our observations, ammonium, guanidinium, and imidazolium systems are primary proton donor groups that govern the structure and assembly phenomena.

The N–H proton donor of the ammonium group forms near-linear hydrogen bonds with very short HB lengths of approx. 1.50 Å (1_2_, 2_2_, and 3_1_; see Tables 2 and S1 in the ESI†), whereas the N–H of histidine is approximately 1.45 Å in length. The HB lengths of the guanidinium residue in the arginine cation 4_2_ are distinctly longer (1.74 Å on average). The non-ionized IBA carboxyl group in binary mixture 4_2_ leads to the formation of a strong hydrogen bond with a length of 1.617 Å (170 deg.), which is very similar to those of 3a and 3b. Most likely, the weakest N–H proton donor of indole to the same betaine proton acceptor forms a bridge with a length of 1.903 Å, which is still much lower than in non-ionized associates.^29^ The methyl substituents of betaine form C–H⋯O hydrogen bonds if spatially close to the carboxylate, where the bond lengths range from 2.30–2.35 Å for 4_2_. Note that the bond lengths for the cyclic structures 1a, 2a, and 4b are less prominent because of common HB geometrical restraints (see Table 2).

**Table 2:** Intra- and intermolecular hydrogen bond parameters calculated for B3LYP/6-31++G(d,p) in equimolar binary mixtures and ion pairs of indole-3-butyric acid and arginine, histidine and betaine in water

No	Total charge [a.u.]	Hydrogen bond	Intermolecular hydrogen bond parameters length (Å), angle (degree)
1a	0	N1^Arg_gua^H⋯O1^IBA^	1.841 (148.3)
		N2^Arg_gua^H⋯O1^IBA^	1.832 (148.1)
		N^Arg_amm^H⋯O1^Arg^	1.830 (122.3)
		N^IBA_Ind^H⋯O1^Arg^ a	1.857 (160.9)
1b	0	N1^Arg_gua^H⋯O1^IBA^	1.790 (164.3)
		N1^Arg_gua^H⋯O2^IBA^	2.552 (106.3)
		N^Arg_amm^H1⋯O1^IBA^	1.663 (175.5)
		N^Arg_amm^H2⋯O1^Arg^ a	1.881 (122.0)
2a	0	N^IBA_Ind^H⋯O1^His^	1.954 (158.2)
		N^His_amm^H⋯O1^His^ a	1.809 (123.8)
		C(O)O1H^IBA^⋯N1^His Imid^	1.709 (163.8)
2b	0	N^His_amm^H⋯O1^His^ a	1.872 (122.0)
		N^His_amm^H⋯O1^IBA^	1.657 (170.4)
		N1^His_Im^H⋯O1^IBA^	1.538 (159.3)
		N1^His_Im^H⋯O2^IBA^	2.911 (151.2)
3a	0	N^Pro_amm^H1⋯O1^IBA^	1.770 (169.2)
		C(O)O1H^IBA^⋯O1^Pro^	1.586 (170.8)
3b	0	N^Pro_amm^H1⋯O1^IBA^	1.532 (175.4)
		C(O)O1H^Pro^⋯O2^IBA^	1.426 (169.0)
3c	0	C(O)O1H^IBA^⋯N^Pro_amm^	1.447 (175.4)
		C(O)O1H^Pro^⋯O2^IBA^	1.603 (168.6)
3_1_	−1	N^Pro_amm^H1⋯O1^IBA^	1.561 (174.9)
		N^Pro_amm^H1⋯O2^IBA^	2.667 (126.6)
		N^Pro_amm^H2⋯O1^Pro^ b	1.978 (119.3)
4a	0	C(O)O1H^IBA^⋯O1^Bet^	1.621 (170.4)
		C(O)O1H^IBA^⋯O2^Bet^	3.081 (133.8)
		N^IBA_Ind^H⋯O2^Bet^	1.923 (161.5)
4b	−1	C^bet2^H⋯O1^IBA^	2.927 (144.1)
		C^Met1^H⋯O1^IBA^	2.934 (141.8)
		C^Met1^H⋯O2^IBA^	2.314 (168.6)
		C^Met2^H⋯O1^IBA^	2.358 (166.5)
		C^Met2^H⋯O1^Bet^ c	2.334 (122.6)
		C^Met3^H⋯O1^Bet^ c	2.262 (123.8)
		N^IBA_Ind^H⋯O2^Bet^	1.860 (166.5)

a Intramolecular hydrogen bond between ammonium group and carboxylate of arginine.

b Intramolecular hydrogen bond between *N*-methyl group and carboxylate of betaine.

c Eutectic.

Tables 3, S2 and S3 in the ESI† present the complexation energy values used to assess the potential proton-donor ability of the investigated zwitterions and betaine during interactions with the IBA anion. The most likely structure for l-histidine is 2b, which exploits both ammonium and imidazolium moieties and forms chelate hydrogen bonds to both carboxylate oxygen atoms, with a total energy of −28.8 kcal mol^−1^. Interestingly, although 1b is topologically similar to 2b, 1b has a relatively low total energy of −11.7 kcal mol^−1^, probably because the hydrogen bonding of the guanidine residue is not very effective (Table 2). The most stable structure among 3a–3c (Table 3) is 3a, a complex of a zwitterion and a non-ionized acid. However, 3b is less stable than 3a by only approximately 2.8 kcal mol^−1^, which demonstrates how electrostatic attractive forces can act in conjunction with two strong proton donors to form close contacts with oxygen atoms of the carboxylate (the complexation energy is estimated to exceed −38 kcal mol^−1^). The interaction of betaine and IBA anion reached a minimum value of −3.3 kcal mol^−1^, where even the N–H of IBA and carboxylate of betaine participate in the total score (Table 3). Tables 3 and S2 (ESI†) present the complexation energy for single, conformationally unrestrained ammonium (tetraalkyl-ammonium for 4_2_) joints, which confirms the following ranking in terms of the hydrogen bond parameters: 2 > 1 > 3 > 4.

**Table 3:** Electronic energy (Hartree) and complexation energy of ionic or neutral components of ion pairs or binary mixtures in water environment for geometries calculated at B3LYP/6-31++G(d,p) level

No.	Total charge [a.u.]	Class of compound	Type of interaction	[Hartree]	Complexation energy [kcal mol^−1^]
1a	0	Protonated amino acid + acid anion	^+^Gua–^−^O_2_C	−1277.0433542^a^	−19.2
1b	0	Protonated amino acid + acid anion	^+^Gua–^−^O_2_C	−1277.0456456^a^	−11.7
			^+^NH_3_–^−^O_2_C		
2a	0	Zwitterion of amino acid + acid	^0^Imid–HO(O)C	−1219.2429738^b^	−13.5
2b	0	Protonated amino acid + acid anion	^+^NH_3_–^−^O_2_C	−1219.2446678^b^	−28.8
3a	0	Zwitterion of amino acid + acid	^+^NH_2_–^−^O_2_C	−1071.6216155^c^^,^^d^	−15.1
			CO_2_^−^–HO(O)C		
3b	0	Protonated amino acid + acid anion	^+^NH_2_–^−^O_2_C	−1071.6171871^c^	−38.4
			C(O)OH–^−^O_2_C		
3c	0	Amino acid + acid	^0^Amino-HO(O)C	−1071.6140875^d^	−28.1
			C(O)OH–OC(OH)		
3_1_	−1	Zwitterion of amino acid + acid anion	^+^NH_2_–^−^O_2_C	−1071.1582504	−14.0
4a	0	Betaine + acid	C(O)OH–^−^O_2_C	−1072.8026406^e^	−14.1
4b	−1	Betaine + acid anion	^+^NR_4_–^−^O_2_C	−1072.3263498^e^	−3.3

a Structure 1a is of 1.44 kcal mol^−1^ less stable than structure 1b.

b Structure 2a is of 1.06 kcal mol^−1^ less stable than structure 2b.

c Structure 3b is of 2.78 kcal mol^−1^ less stable than 3a.

d Structure 3c is of 4.72 kcal mol^−1^ less stable than 3a.

e Structure energies of 4a and 4b cannot be compared due to the difference in overall structure charge.

Note that betaine carboxylate is a weaker proton acceptor for IBA acid than IBA carboxylate is for the ammonium ion in 4_2_. Moreover, our calculations confirm that although the structures of protonated l-arginine or l-histidine, l-proline, and betaine form low-barrier hydrogen bonds as zwitterions, the ionic character of these structures is maintained in water. Ionic pairs are formed with the IBA anion, where the 1 : 1 complexes can be considered as ammonium salts, except for products 3 and 4. The structure of 4_2_ confirms that glycine-betaine, and betaines in general, retain their ionic nature, making it impractical to modify betaines to protic ionic liquids, at least by using aliphatic acids. Product 3 containing l-proline and IBA is most likely a binary mixture. Therefore, molecular calculations preliminarily confirm that products 1 and 2 are ammonium salts, whereas 3 and 4 are binary mixtures.

The UV spectra showed that IBA was present in all products 1–5 and that the IBA form did not appear to affect the wavelength at which maximum absorption occurred. The IBA maximum absorption (*λ*_max_) at 221 and 280 nm in aqueous solution was observed for anionic IBA, as a component of a mixture or as a pure substance. These results are in agreement with previous reports of no change in *λ*_max_ for either ascorbic acid or its anion.^30^

The ^1^H and ^13^C NMR spectra were analysed to identify changes in the chemical shifts of the products relative to the initial substrates. Chemical shifts in the ^1^H NMR spectrum can be used to identify ionic or acidic forms of IBA. The shifts in the signal values derived from alpha hydrogen atoms change from approx. 2.43 ppm for the IBA acid form to approx. 2.30 ppm for the ionic form. This result implies an anionic form for IBA for products 1, 2, and 5. The signals from the hydrogen atoms in l-arginine or l-histidine for products 1 and 2 were shifted relative to the pure amino acid by approx. 0.01–0.50 ppm.

**Fig. 2:**
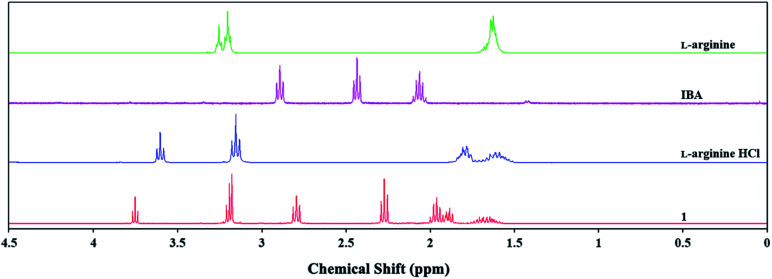
Influence of IBA and HCl on chemical shifts of l-arginine cation in ^1^H NMR spectrum.

Similar chemical shifts were also observed for l-arginine hydrochloride or l-histidine hydrochloride, which has been reported in the literature to indicate the cationic form.^31,32^ The abovementioned shifts are shown in Fig. 2 and S17 (ESI†) for 1 and 2, respectively. Spectroscopic studies were used to observe chemical shifts in the ^13^C NMR spectrum of the l-arginine (1) or l-histidine (2) cation corresponding to the pure amino acid and its hydrochloride (Fig. S16 and S18 in the ESI†). The spectroscopic data and molecular calculations support the hypothesis that products 1 and 2 were ammonium salts, whereas IBA was in anionic form.

**Fig. 3:**
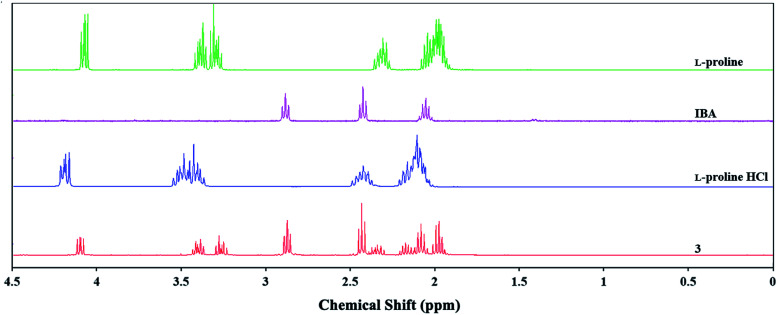
Influence of IBA and HCl on chemical shifts of l-proline in ^1^H NMR spectrum.

However, no significant chemical shifts in the ^1^H and ^13^C NMR spectra of product 3 were observed relative to l-proline (Fig. 3 and S19 in the ESI†). By contrast, chemical shifts of approx. 0.13 ppm were observed for signals originating from the hydrogen atoms in the l-proline hydrochloride spectrum relative to l-proline and product 3. The absence of chemical shifts for binary mixtures of compounds containing nitrogen atoms and carboxyl groups together with signal shifts of approximately 0.01 ppm imply that these compounds contain intermolecular hydrogen bonds.^33,34^ Considering the molecular calculation results, it can be assumed that the reaction of l-proline and IBA produces a binary mixture in a 1 : 1 molar ratio. However, product 3 may partially exist as an ion pair in aqueous solution.

**Fig. 4:**
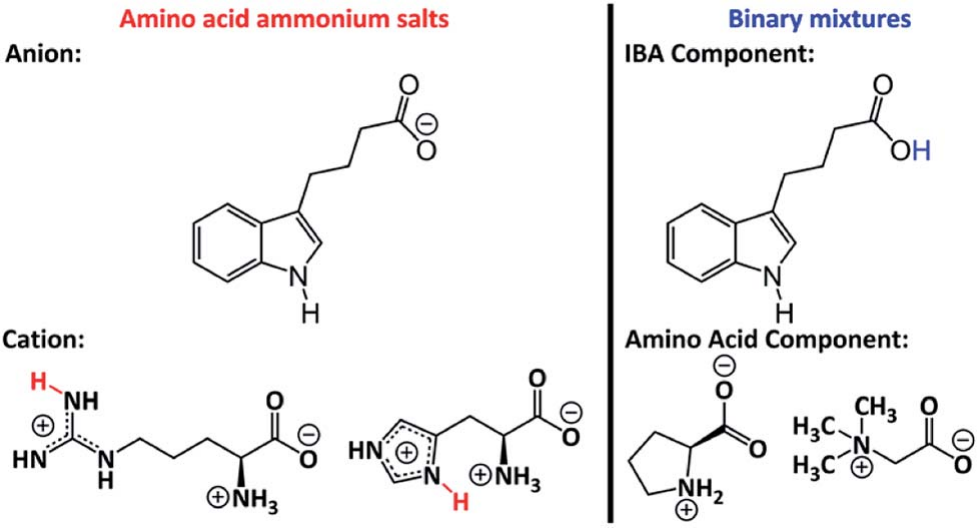
Structures of obtained ammonium salts (1 and 2) and binary mixtures (3 and 4).

An analysis of the data obtained for product 4 and literature reports led us to expect that reacting glycine betaine with IBA would not produce an ammonium salt. In 2018, a non-ionic product of the reaction of glycine betaine with a carboxylic acid was reported, which is attributed to proton transfer between carboxyl groups. This effect results from the considerably higher bond strength between hydrogen and oxygen in the carboxylic acid (that is, carboxylic acid has a higher p*K*_a_ than glycine betaine).^35^ As the p*K*_a_s of IBA and pelargonic acid are 4.70 and 4.95, respectively, it was speculated that a similar phenomenon could occur. The ^1^H NMR and ^13^C NMR spectra for glycine betaine and product 4 confirmed this assumption. There were no changes in the chemical shifts for product 4 relative to glycine betaine (Fig. S20 and S21 in the ESI†). Thus, the abovementioned results and the molecular calculation results show that product 4 was a binary mixture. Note that the resulting binary mixtures 3 and 4 may have been eutectics, but further research is needed to confirm this speculation. The product structures are shown in Fig. 4.

Table 1 present thermal stability and phase transformation results, which show melting points of 181 °C, 110 °C, and 112 °C for ammonium salts 1 and 2 and binary mixture 3, respectively. Salt 2 was the only compound with a crystallization temperature (*T*_c_ = 93 °C) within the investigated temperature range. Only products 3, 4, and 5 exhibited glass transition temperatures (*T*_g_ = 13 °C, *T*_g_ = 2 °C, and *T*_g_ = −18 °C, respectively). Therefore, it was concluded that converting an amino acid (l-arginine – *T*_m_ = 222 °C; l-histidine – *T*_m_ = 282 °C) into an ammonium salt reduces the melting point because of the increased asymmetry in the structures of the obtained salts.^36,37^ However, the melting point of the synthesized binary mixtures depends on the components as well as the type and strength of the intervening hydrogen bonds.^38^ Note that IBA combined with amino acids has a significantly higher thermal stability than the acid form of IBA. By comparison, the thermal stability of IBA combined with choline or glycine betaine is considerably less enhanced. This difference is related to the thermal stability of the cation or component of the binary mixture used to synthesize a given product.

The solubility test results (Table S4 in the ESI†) indicate limited water solubility of the synthesized products 1–4 and poor water solubility for IBA. By contrast, ionic liquid 5 and the potassium salt of IBA were highly soluble in water.^26,39^ Thus, leaching of these products into deep layers of soil would be mitigated were these substances released into the environment. However, the aforementioned water solubilities are sufficient for application purposes.^40^ Converting IBA into a salt or a binary mixture form reduced the IBA solubility in DMSO, acetonitrile, and chloroform. The largest differences in solubility were observed for methanol and acetone. Products 3, 4, and 5 had the same solubility in methanol and acetone as IBA, whereas products 1 and 2 had reduced solubility.

**Fig. 5:**
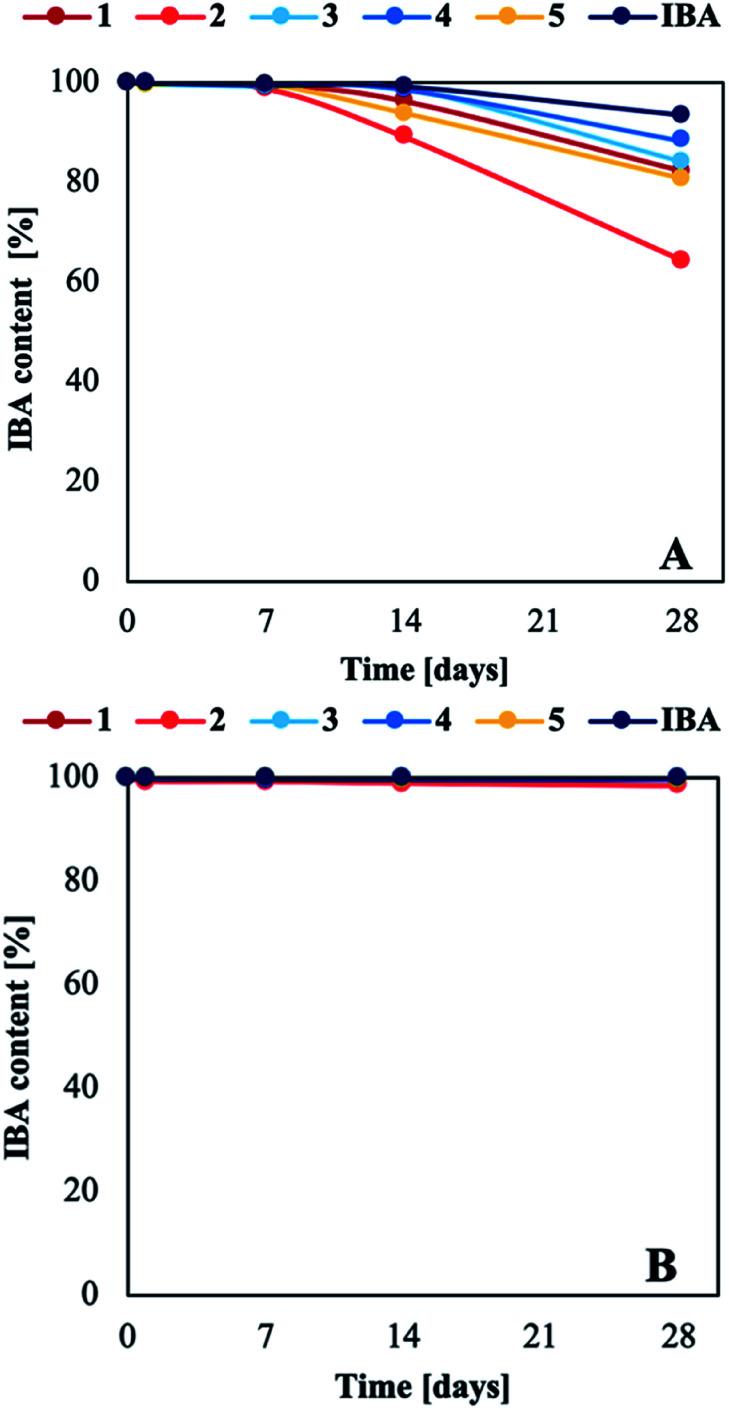
Change in IBA (acid or anion) content in aqueous solutions stored at constant light intensity (A) and in the dark (B).

Indole-3-butyric acid is known to be the most stable form of natural auxins; however, like tryptophan (Trp), indole-3-acetic acid (IAA), and indole-3-propionic acid (IPA), IBA is degraded aerobically depending on many factors, including the oxygen level and exposure to heat and light.^41–44^ In this study, we determined how light accelerated the abovementioned decomposition (the methodology is described in the ESI†). The intensity of the maximum absorption for IBA in acid or anionic forms (*λ*_max_ = 221 nm) decreased after only 7 days in prepared water solutions exposed to stable visible light (Fig. 5A and Table S5 in the ESI†). By contrast, the aqueous solutions stored in the absence of light were stable over the entire duration of the experiment – 28 days (with the exception of salt 2, which degraded by approx. 1% (Fig. 5B and Table S7 in the ESI†)). The solutions changed from colourless to dark yellow during testing, and a solid precipitate was observed for samples exposed to light (see Tables S6 and S8 in the ESI†). The decomposition products of Trp, IAA, and IPA are kynurenic acid (KYN) or KYN derivatives, which have been reported to produce a yellow or brown colouration in aqueous solutions, and a brown solid precipitate has been observed at high concentrations. As both acidic and ionic forms of IBA degrade, the likely degradation product is 5-(2-formamidophenyl)-5-oxopentanoic acid, a KYN derivative.^41–44^

The light stability of the aqueous solutions was strongly correlated with the form of IBA and the type of cation or second binary mixture component in the products. Products 1, 2, and 5 were less stable than IBA and binary mixtures 3 and 4 under light exposure. After 28 days, the IBA concentration in aqueous solutions of ammonium salts decreased by 18% (1), 36% (2), and 20% (5), compared to a decrease of approx. 15% for the binary mixtures, and of only 7% for IBA in acid form (see Tables S4 and S6 in the ESI†). These results show that the anionic form of IBA is less stable than the acid form. This phenomenon may be related how organic cations and a negative charge affect the electron distribution in the IBA structure. The largest decrease in the absorbance intensity was observed for salt 2, which was most likely caused by the parallel photodegradation of l-histidine,^45,46^ assuming that the other components did not photodegrade under the same conditions.^18,47–51^

**Fig. 6:**
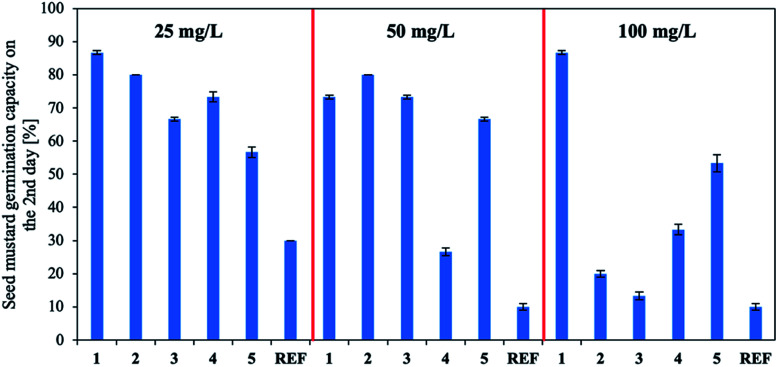
Effect of IBA concentration on mustard seed germination capacity on second day.

Finally, the IBA biological activity was determined. Three concentrations of the active substance were used for biological tests (the methodology is described in the ESI†): the IBA concentration recommended by the manufacturer (50 mg L^−1^) and half and twice this concentration (25 mg L^−1^ and 100 mg L^−1^, respectively). The quantity of plant growth hormones used was correlated with the stage of plant growth and the time over which the plant interacted with the solution.^26^ A commercial preparation containing IBA (Rhizopon AA – REF) was used in the control test. The results (Fig. 6 and Table S9 in the ESI†) indicate that aqueous solutions of products 1–5 stimulated mustard seed germination overall and notably accelerated germination compared to the reference (for which germination remained at 10–30%), especially at the lowest concentration (25 mg L^−1^). All investigated concentrations of product 1 highly stimulated seed germination (80–90%). A similar trend was observed for compound 5, although germination was lower (50–70%). Using compounds 2 and 3 at the highest tested concentration (100 mg L^−1^) decreased germination (to 10–20%). Using product 4 at the recommended dose (50 mg L^−1^) decreased germination (to approx. 30%) was observed. Note the use of the products shortened the germination time.

**Fig. 7:**
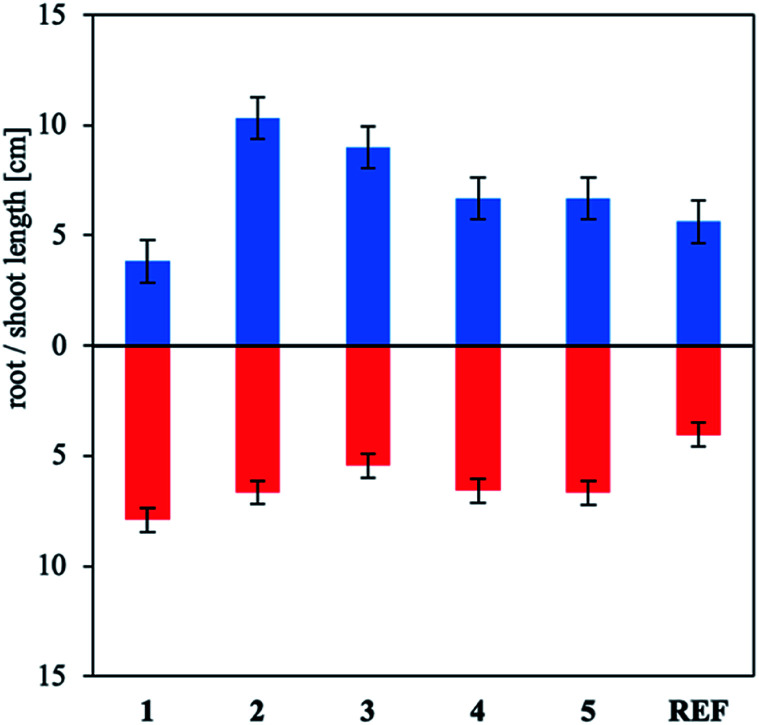
Effect of ammonium salts and binary mixtures containing 25 mg L^−1^ indole-3-butyric acid on mustard shoot development and root length.

To supplement the germination study, the effect of the products on plant growth and the root length was assessed. Using the synthesized products at concentrations of 25 and 50 mg L^−1^ (Fig. 7 and S22 in the ESI†) increased the development of the root system and plant shoots. Very interesting results were obtained for 1, which expanded the root system while inhibiting shoot development. This effect decreases plant resistance to external factors and is therefore shoot growth by 15%, whereas all the other compounds stimulated plant development. Use of all compounds at 100 mg L^−1^ concentrations reduced plant development (Fig. S23 in the ESI†). This effect was most notable for 3, for which plant growth was completely inhibited desirable.^52^ The 50 mg L^−1^ solution of binary mixture 4 inhibited.

The ionic form of IBA stimulated root system growth noticeably more than the other IBA forms, irrespective of the concentration used. However, the growth of the aboveground part of the plant was considerably more sensitive to the type of counterion or the second component of the binary mixture. Note that similar results have been reported for applying IBA to other plants to stimulate germination.^53,54^

## Conclusions

Novel bioammonium salts and natural binary mixtures containing IBA as an anion or as a component of the binary mixture, as well as various amino acids, glycine betaine and choline, were characterized in this study. The synthesis methodology yielded products at high yields and purities. Molecular calculations and UV-Vis, ^1^H and ^13^C NMR spectra were used to confirm the identity of the products containing l-arginine and l-histidine cations, which were ammonium salts, as well as two l-proline and betaine products containing non-ionic IBA. The correlation between the computational data and the NMR spectra indicated that ionic bonding affects chemical transfer significantly more than hydrogen bonding. The results of solubility tests showed that the chemical structure of the cation or the binary mixture component influenced the affinity of IBA towards organic solvents. The faster decomposition of the IBA anion compared to the IBA acidic form under light may decrease the effectiveness of IBA agrochemical applications. Therefore, applying IBA in the form of a binary mixture may resolve problems with applying IBA in the field. Biological studies confirmed that the ammonium salts and binary mixture containing IBA stimulated the efficiency and rate of seed germination. Depending on the compound and the concentration used, 30 to 80% of the seed germinated on the second day. In addition, the application of 25 and 50 mg L^−1^ ammonium salts containing l-histidine, choline cations and anionic IBA increased root and shoot development, implying that the ionic form of IBA stimulates plant growth. In conclusion, characterizing the form in which a biologically active ingredient occurs is essential for determining how light affects the stability of the structure and biological activity.

## Conflicts of interest

There are no conflicts to declare.

## Supplementary Material

RA-010-D0RA09136G-s001
