# Supplementary material for: Use of ammonium salts or binary mixtures derived from amino acids, glycine betaine, choline and indole-3-butyric acid as plant regulators
Source: RSC Adv. 2020 Nov 26;10(70):43058–65. doi: 10.1039/d0ra09136g (PMC9058124; doi:10.1039/d0ra09136g)
Supplement: RA-010-D0RA09136G-s001 [file RA-010-D0RA09136G-s001.pdf]

Electronic Supplementary Material (ESI) for RSC Advances.
This journal is © The Royal Society of Chemistry 2020

Use of ammonium salts or binary mixtures derived from amino acids, glycine betaine, choline and indole-3-butyric acid as plant regulators

Damian Krystian Kaczmarek,[a] Anna Parus,[a] Marek Łożyński[a] and Juliusz Pernak[a,*]

[a.] Faculty of Chemical Technology, Poznan University of Technology, Berdychowo 4, Poznan 60-965, Poland

* Corresponding author. E-mail: juliusz.pernak@put.poznan.pl

Experimental

Materials

Glycine betaine ([BET], purity ≥98%, CAS Number 107-43-7), L-histidine ([HIS], purity ≥99%, CAS Number 71-00-1), L-arginine ([ARG], purity ≥98%, CAS Number 74-79-3), L-proline ([PRO], purity 99%, CAS Number 147-85-3), choline chloride (99%, CAS Number 67-48-1) and solvents (methanol, DMSO, acetonitrile, acetone, isopropanol, ethyl acetate, chloroform, toluene, hexane) were purchased from Sigma–Aldrich and used without further purification. Indole-3-butyric acid (IBA, purity 98%, CAS Number 133-32-4) was supplied by Alfa Aesar. Dowex-Monosphere 550A anion exchange resin (OH) was purchased from Sigma–Aldrich. Water for solubility was deionized with the conductivity below 0.1 mS cm^{-1} from demineralizer HLP Smart 1000 (Hydrolab).

Synthesis

The respective amino acids or glycine betaine (0.05 mol) were dissolved in 50 mL of solvents (water:ethanol in a 10:1 v/v ratio). Indole-3-butyric acid (IBA; 0.05 mol) was then slowly introduced into the reaction vessel. The reaction was conducted at ambient temperature (40 °C) for 24 h in the dark. The solvent was allowed to evaporate from the open vessel for over 48 h, and the resulting mixture was dried in a laboratory dryer under reduced pressure at 60 °C for 24 h. The final products were stored under vacuum over P_4O_{10} in the absence of light.

The synthesis of the ionic liquid with the choline cation was carried out according to the synthesis method described in the literature.[1]

General

NMR spectra (^{1}H and ^{13}C NMR) were prepared in deuterated water and methanol (4:1) containing TMS as the internal standard. Measures were carried out using a Varian Mercury 300 and Varian VNMR-S 400 MHz spectrometers.

The UV spectra of the samples were recorded at 25 °C using a UV-1601 spectrophotometer (Rayleigh) in the 200-360 nm range using quartz cuvettes with a

1-cm path length and a 1.5-nm bandwidth. Water solutions of the products at concentrations of 0.00247 mmol L^{-1} were loaded into individual cuvettes and analysed (distilled water was used as the reference).

The water content was determined using an Aquastar volumetric Karl Fischer titrator EMD Millipore (Billerica, MA, USA) with a Composite 5 solution as the titrant and anhydrous methanol as the solvent.

Thermal gravimetric analysis (TGA) was performed using a Mettler Toledo Star^e TGA/DSC1 unit (Leicester, UK) under nitrogen. Samples weighing between 2 and 10 mg were placed in aluminum pans and heated from 30 to 450 °C at a heating rate of 10 °C min^{-1}.

The thermal transition temperature was determined by differential scanning calorimetry (DSC) with a Mettler Toledo Star^e DSC1 (Leicester, UK) unit under nitrogen. Samples weighing between 5 and 15 mg were placed in aluminium pans and heated from 25 to 120 °C at a heating rate of 10 °C min^{-1}, cooled with an intracooler at a cooling rate of 10 °C min^{-1} to −100 °C, and then re-heated to 140 °C.

The pH of the water solutions of the obtained salts and binary mixtures (in a 10:0.1 mass ratio) was measured at 25 °C using a S47 SevenMulti™ Mettler Toledo equipped with a Mettler Toledo semi-micro combination electrode (Inlab® 421) filled with 3 M KCl.

Chemical stability

The chemical stability of the products was determined following a previously reported methodology.[2] The stability of products **1**, **2**, **3**, **4** and **5** and indole-3-butyric acid (IBA) was estimated for 0.00247 mol L^{-1} aqueous solutions (corresponding to an IBA anion concentration of 0.05% m/v). First, 0.0494 mmol of the selected compounds or binary mixtures were dissolved in 20 mL of water and inserted into sealed glass vials containing a magnetic stir bar. All the resulting solutions were mixed using a Heidolph MR Hei-End stirrer equipped with a heat-on anodized block at a constant temperature of 25.0 °C (with an accuracy of ± 0.1 °C) in the dark or under a steady luminous intensity (luminous flux: 1060 lm, light source colours: 4100 K, general colour rendering index: Ra ≥80). After a period of

time, a 0.02 mL sample was collected from each vial and diluted 75-fold. The absorbance of the sample solutions was measured at λ_{max} = 221 nm for anionic IBA and IBA using a Rayleigh UV-1800 spectrophotometer. The concentrations of the compounds in water were determined based on calibration curves of the absorbance *vs* the concentration for each substance. The presented results are the average of three separate measurements.

Solubility

The solubility of products obtained as well as IBA was determined according to previously described methodology.[3] Commonly used solvents were selected for the solubility test and ranked in descending order of their Snyder polarity index value (water, 9.0; methanol, 6.6; DMSO, 6.5; acetonitrile, 6.2; acetone, 5.1; ethyl acetate, 4.4; chloroform, 4.1; toluene, 2.3, and hexane, 0.0). The sample of compounds or binary mixtures (0.1 g) was introduced into vials, and then a specific volume of solvent was added. Three types of behaviour were recorded depending on the volume of solvent used. The term 'good solubility' applies to compounds or binary mixtures that dissolved in 1 mL of the solvent. The expression 'limited solubility' indicates that 0.1 g was dissolved in 3 mL of the solvent. The term 'poor solubility' symbolizes that salts, binary mixtures or IBA could not be dissolved in 3 mL of the solvent. All the analyses were conducted at 25 °C.

Molecular Modelling

The initial Cartesian coordinates of the IBA and IBA anions were determined using data from the literature, and representative structures of L-arginine, L-histidine, L-proline and glycine betaine were selected from a set of possible conformers to maximize the interaction between the polar groups of a given ammonium salt or binary mixture. The preliminary stage of optimization calculations in vacuum was omitted to conserve the initial ionic character and charge distribution of the structures. Reliable energy data for individual structures were obtained by a two-stage geometrical optimization using DFT[4] in an aqueous environment with a polarized continuum model, where the method was first applied at the B3LYP/6-31G(d) level and then at the B3LYP/6-31++G(d, p) level.[5-9] The energies of the ionic component interactions for the calculated aggregates in an aqueous environment were determined without considering the error in the superposition of the base (BSSE) because of Gaussian 09[10]

limitations. Individual ion pairs were optimized without constraints using the Gaussian09 program[10] with the LOOSE convergence criteria.

Germination test

Germination tests were performed using a commercial Phytotoxkit test (Tigret Company, Belgium). Garden soil (100 g) was placed on a Phytotoxkit plate, to which 23 mL of water were added. Then, 10 of mustard (*Sinapis alba*) seeds were placed on the plate. The mustard seeds were soaked in an aqueous solution at the respective concentration (25, 50 or 100 mg L^{-1}) for 12 h before being placed in the soil. Reference samples were prepared using a commercial rooting agent at the respective concentration (25, 50 or 100 mg L^{-1}). The prepared Phytotoxkit plates were placed in an incubator at a constant temperature (21 ± 1 °C). The tests were performed in triplicate for each concentration. Observations were made for 7 days. The number of germinated seeds were counted every day and used to determine the germination capacity. The germination capacity was determined using the following formula:

$$G = \left(\frac{Gs}{G}\right) * 100\%$$

Gs – number of germinated seeds, G – total number of examined seeds

The length of roots and shoots was measured at the end of the experiment.

Figure S1. ^{1}H NMR spectrum of L-arginine indole-3-butyrate (**1**)

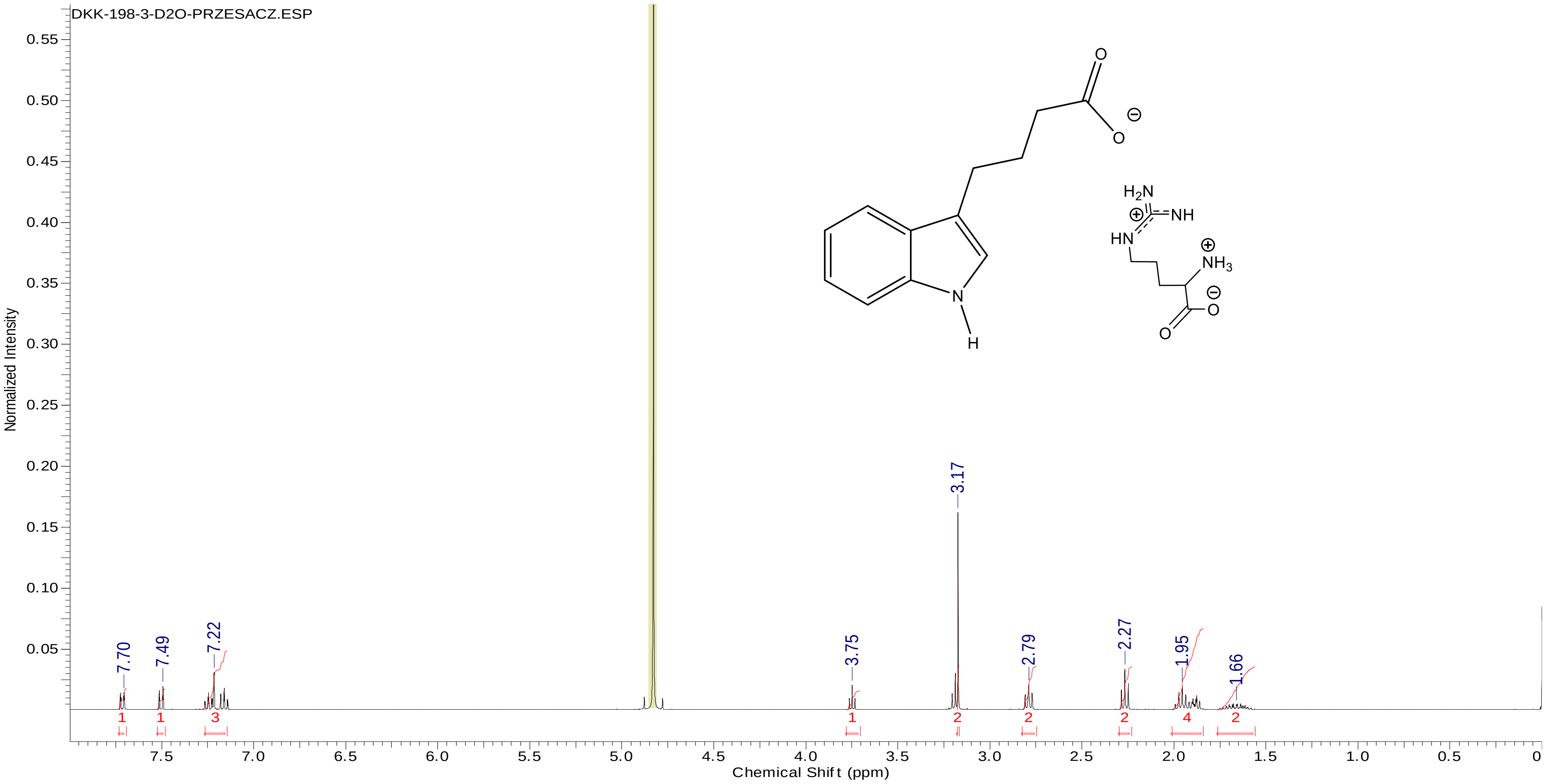

^{1}H NMR (400 MHz, $CD_3OD:D_2O$ 4:1) δ [ppm] = 1.66 (2H, m, CH2C<u>H</u>2CH2CHNH2); 1.95 (4H, m, CH2CH2C<u>H</u>2CHNH2; CH2C<u>H</u>2CH2COO⁻); 2.27 (2H, m, CH2CH2C<u>H</u>2COO⁻); 2.79 (2H, m, C<u>H</u>2CH2CH2COO⁻); 3.17 (2H, m, C<u>H</u>2CH2CH2CHNH2); 3.75 (1H, m, CH2CH2CH2C<u>H</u>NH2); 7.16 (1H, m, CC<u>H</u>NH); 7.22 (2H, m, CCHC<u>H</u>C<u>H</u>CHC); 7.49 (1H, m, CCHCHCHC<u>H</u>C); 7.70 (1H, m, CC<u>H</u>CHCHCHC).

Figure S2. ^{13}C NMR spectrum of L-arginine indole-3-butyrate (**1**)

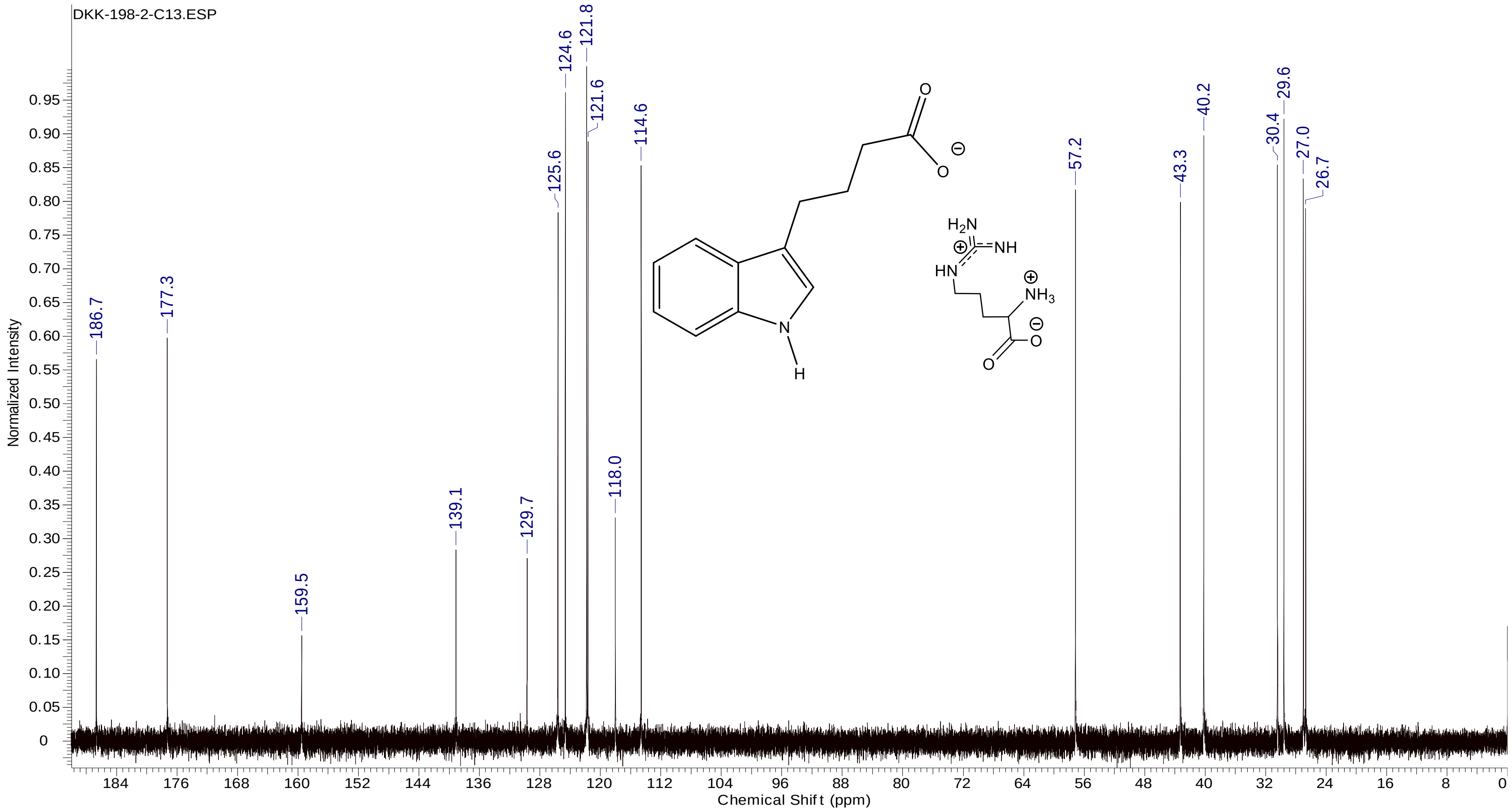

^{13}C NMR (100 MHz, CD_3OD:D_2O 4:1) δ [ppm] = 26.7; 27.0; 29.6; 30.4; 40.2; 43.3; 57.2; 114.6; 118.0; 121.6; 121.8; 124.6; 125.6; 129.7; 139.1; 159.5; 177.3; 186.7.

Figure S3. UV spectrum of L-arginine indole-3-butyrate (**1**)

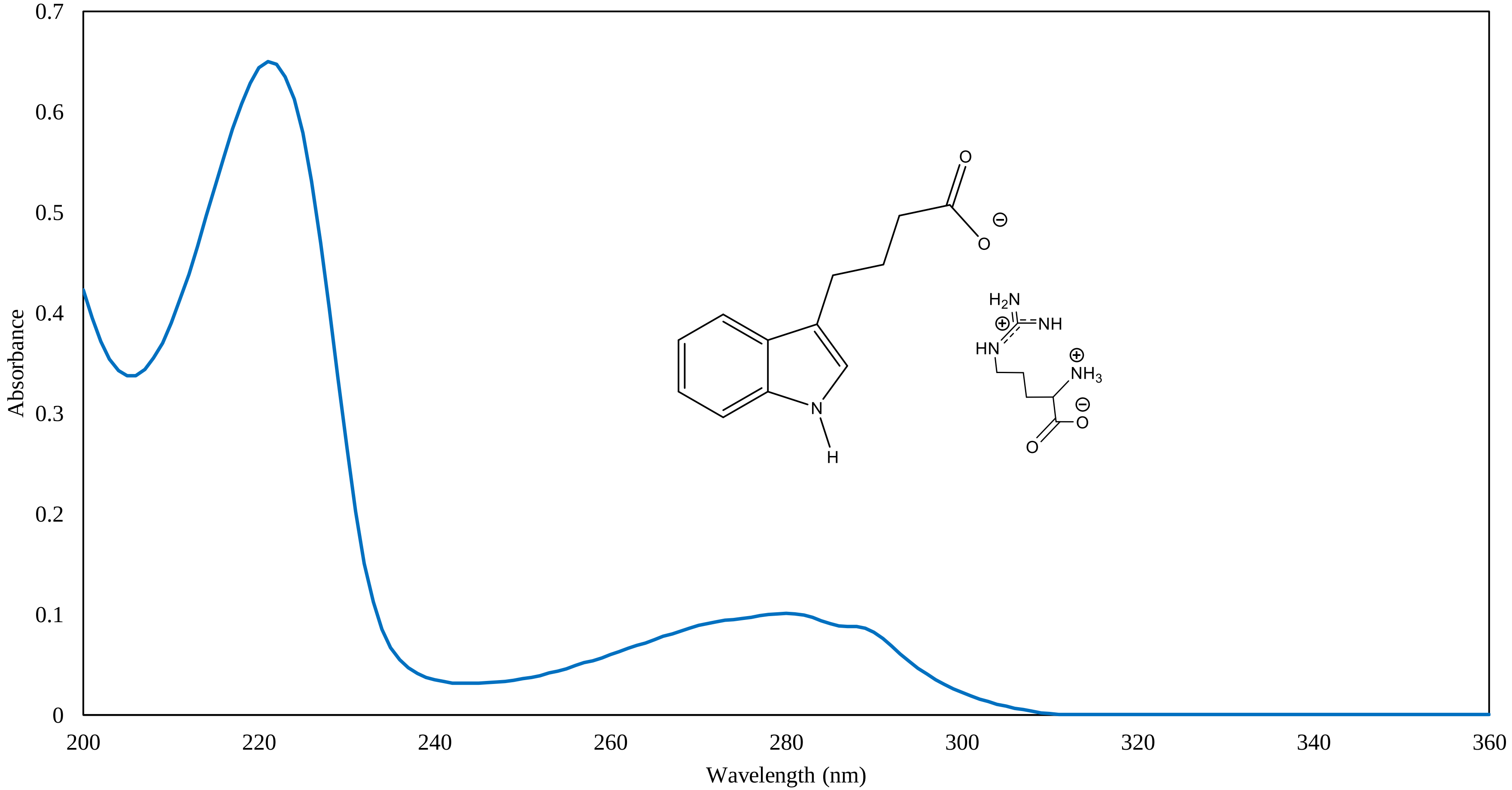

λ_{max} (Water) = 221 nm; $\varepsilon_{\lambda max}$ = 2.63·10^2 dm^3 mol^{-1} cm^{-1}

λ_{max} (Water) = 280 nm; $\varepsilon_{\lambda max}$ = 0.41·10^2 dm^3 mol^{-1} cm^{-1}

Figure S4. ^{1}H NMR spectrum of L-histidine indole-3-butyrate (**2**)

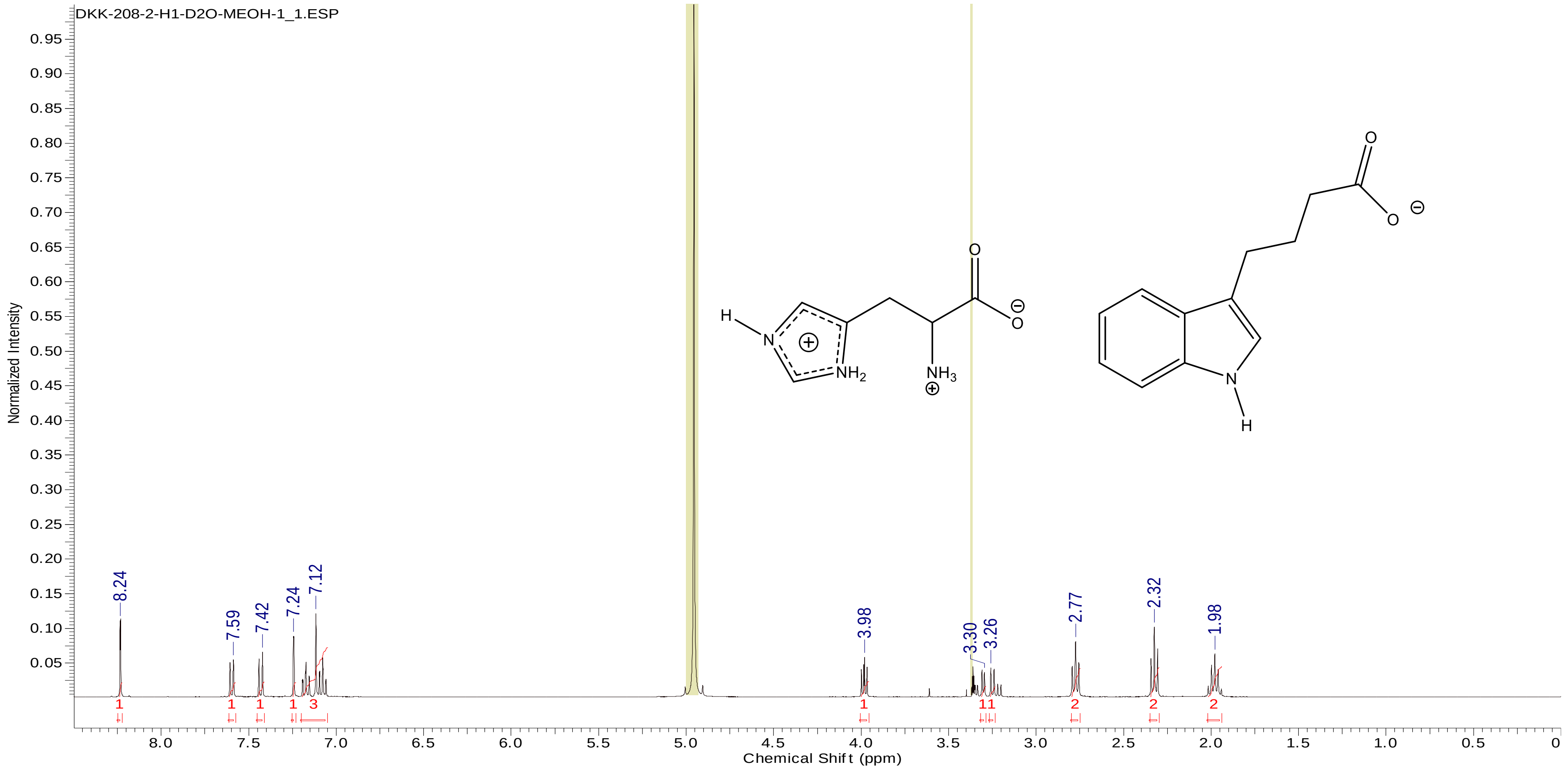

^{1}H NMR (400 MHz, $CD_3OD:D_2O$ 4:1) δ [ppm] = 1.98 (2H, m, $CH_2C\underline{H_2}CH_2COO^-$); 2.32 (2H, m, $CH_2CH_2C\underline{H_2}COO^-$); 2.77 (2H, m, $C\underline{H_2}CH_2CH_2COO^-$); 3.26 (1H, m, $C\underline{H_2}CH(NH_2)COOH$); 3.30 (1H, m, $C\underline{H_2}CH(NH_2)COOH$); 3.98 (1H, m, $CH_2C\underline{H}(NH_2)COOH$); 7.12 (3H, m, $CCHC\underline{H}C\underline{H}CHC$, $CC\underline{H}NH$); 7.24 (1H, s, $CC\underline{H}NHCHN^+H$); 7.42 (1H, m, $CCHCHCHC\underline{H}C$); 7.59 (1H, m, $CC\underline{H}CHCHCHC$); 8.24 (1H, s, $CCHNHC\underline{H}N^+H$).

Figure S5. ^{13}C NMR spectrum of L-histidine indole-3-butyrate (**2**)

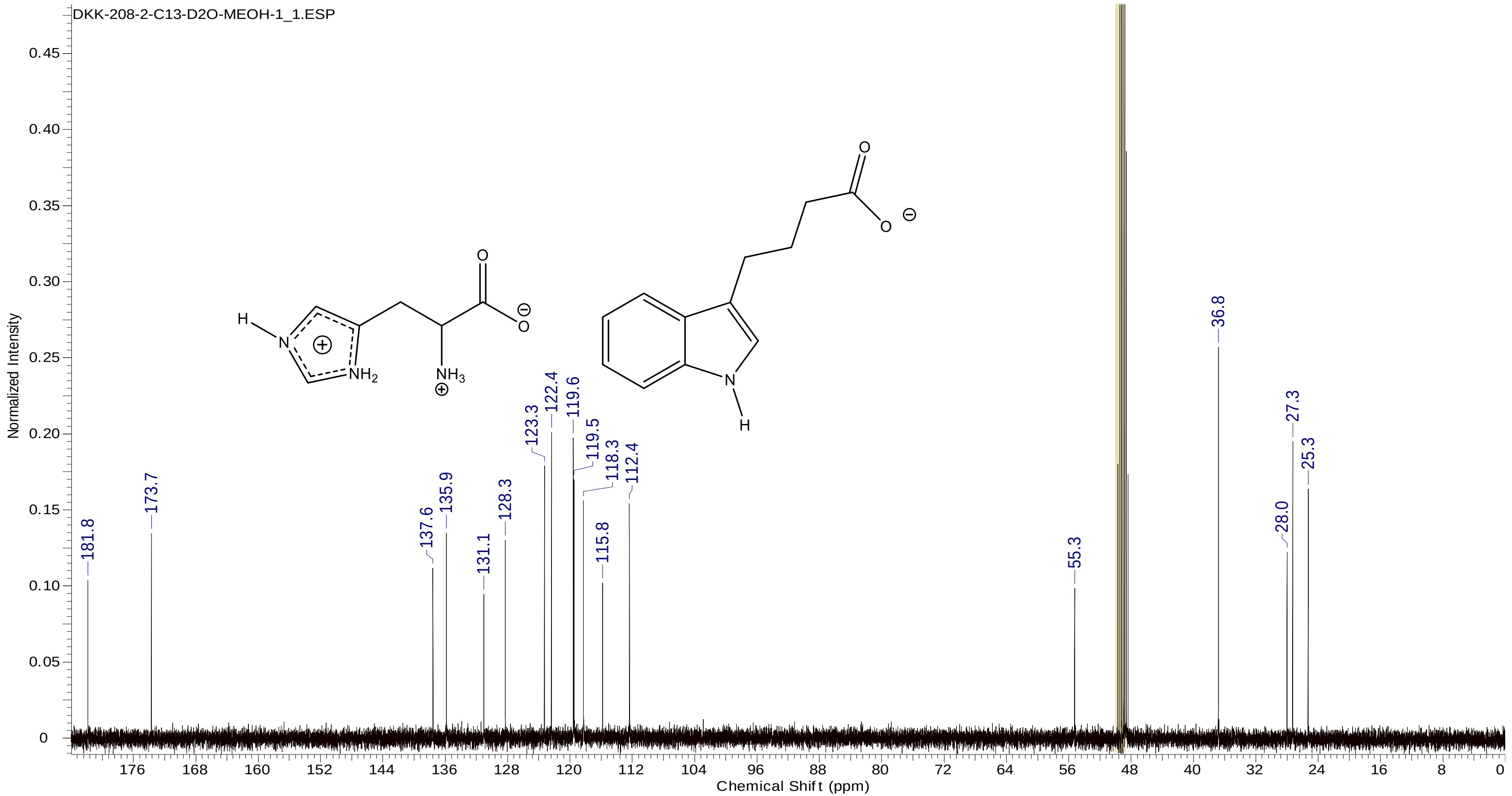

^{13}C NMR (100 MHz, $CD_3OD:D_2O$ 4:1) δ [ppm] = 25.3, 27.3, 28.0, 36.8, 55.3, 112.4, 115.8, 118.3, 119.5, 119.6, 122.4, 123.3, 128.3, 131.1, 135.9, 137.6, 173.7, 181.8.

Figure S6. UV spectrum of L-histidine indole-3-butyrate (**2**)

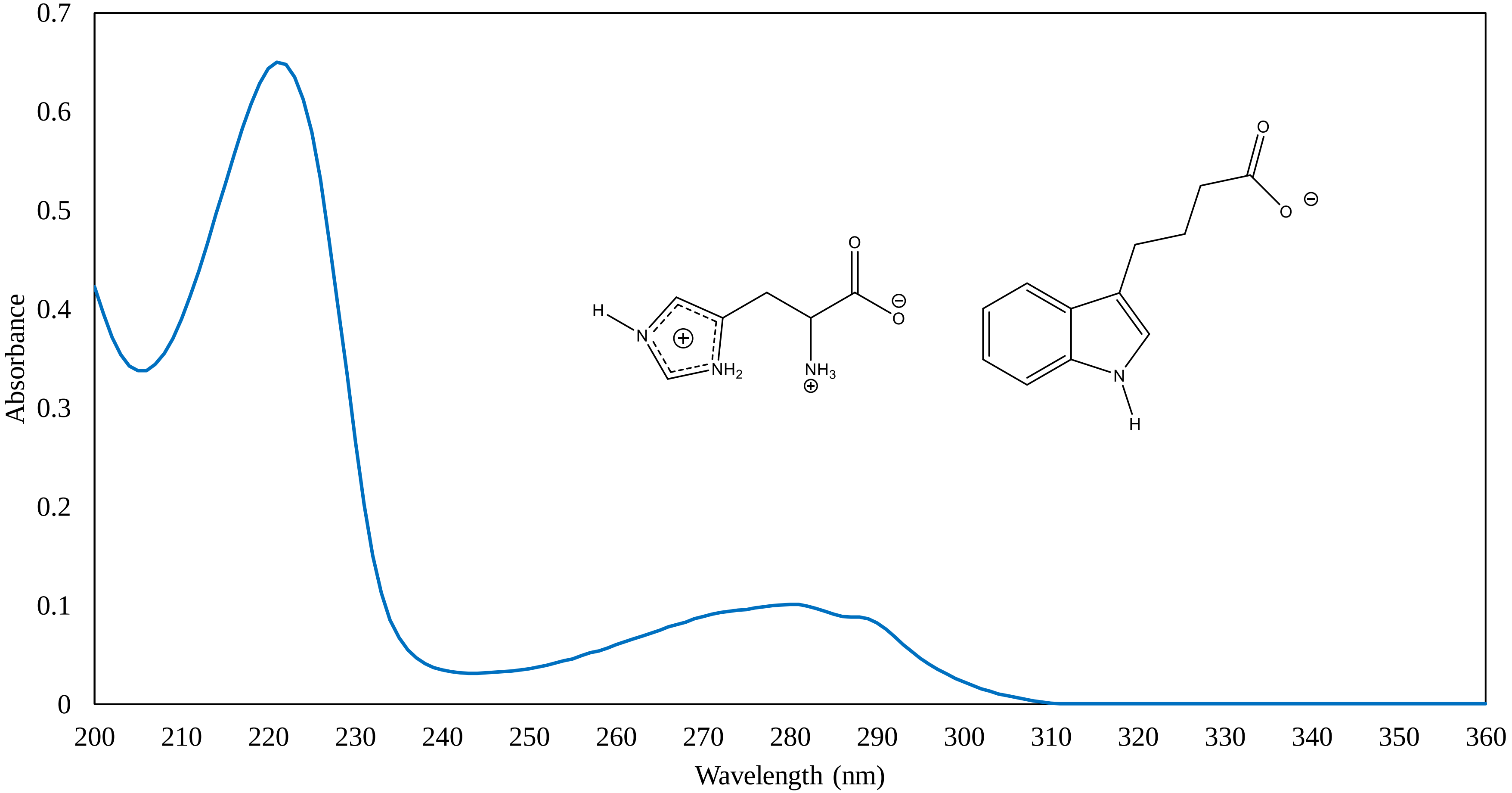

λ_{max} (Water) = 221 nm; $\varepsilon_{\lambda max}$ = 2.88·10^2 dm^3 mol^{-1} cm^{-1}

λ_{max} (Water) = 280 nm; $\varepsilon_{\lambda max}$ = 0.44·10^2 dm^3 mol^{-1} cm^{-1}

Figure S7. ^{1}H NMR spectrum of binary mixture of L-proline:indole-3-butyric acid (1:1) (**3**)

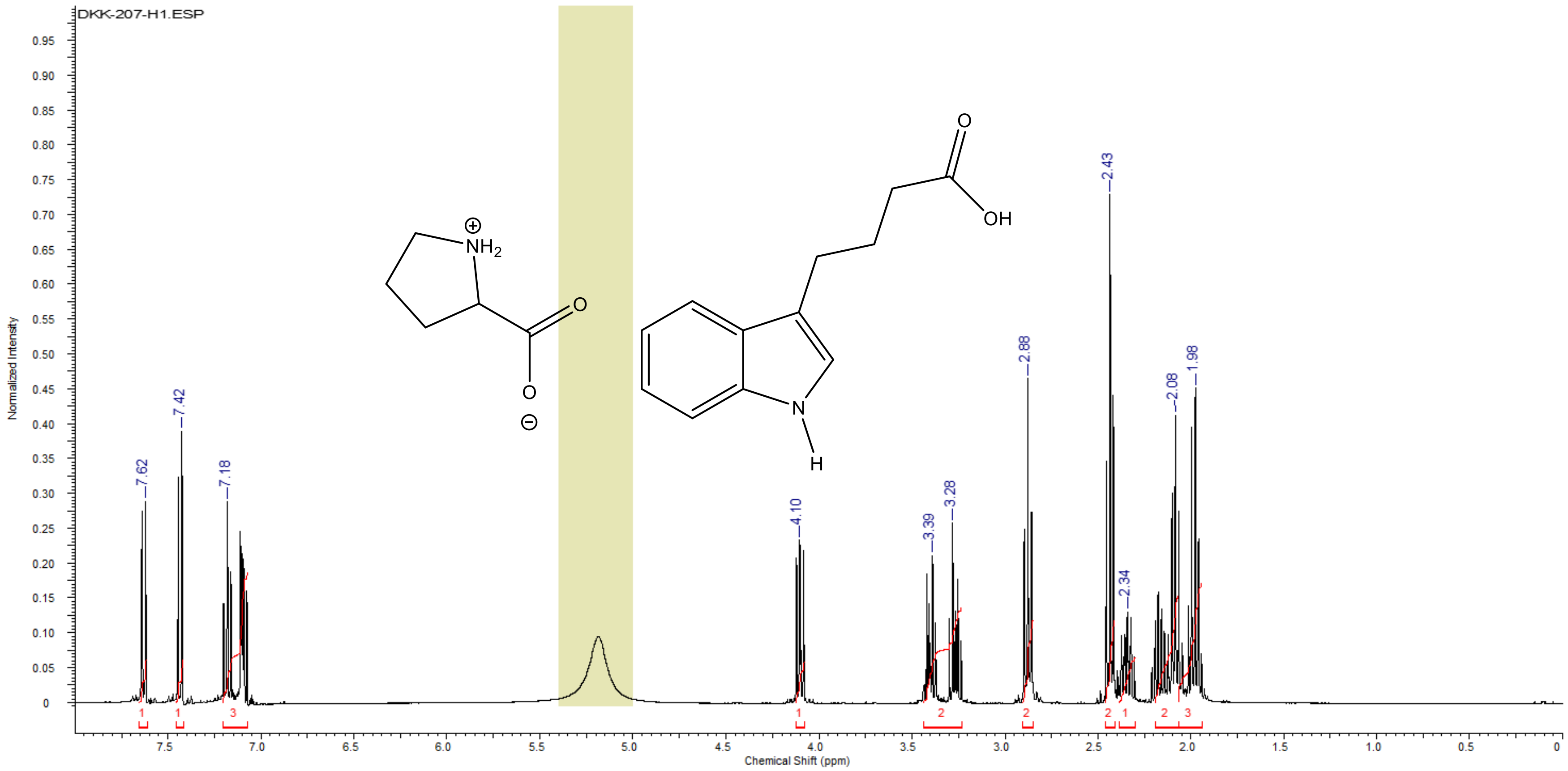

^{1}H NMR (400 MHz, $CD_3OD:D_2O$ 4:1) δ [ppm] = 1.98 (2H, m, $NHCH_2CH_2C\underline{H}_2CHCOOH$, $NHCH_2C\underline{H}_2CH_2CHCOOH$); 2.08 (2H, m, $CH_2C\underline{H}_2CH_2COOH$); 2.34 (1H, m, $NHCH_2CH_2C\underline{H}_2CHCOOH$); 2.43 (2H, m, $CH_2CH_2C\underline{H}_2COOH$); 2.88 (2H, m, $C\underline{H}_2CH_2CH_2COOH$); 3.28 (1H, m, $NHC\underline{H}_2CH_2CH_2CHCOOH$); 3.39 (1H, m, $NHC\underline{H}_2CH_2CH_2CHCOOH$); 4.10 (1H, m, $NHCH_2CH_2CH_2C\underline{H}COOH$); 7.18 (3H, m, $CCHC\underline{H}C\underline{H}CHC$, $CC\underline{H}NH$); 7.42 (1H, m, $CCHCHCHC\underline{H}C$); 7.62 (1H, m, $CC\underline{H}CHCHCHC$).

Figure S8. ^{13}C NMR spectrum of binary mixture of L-proline:indole-3-butyric acid (1:1) (**3**)

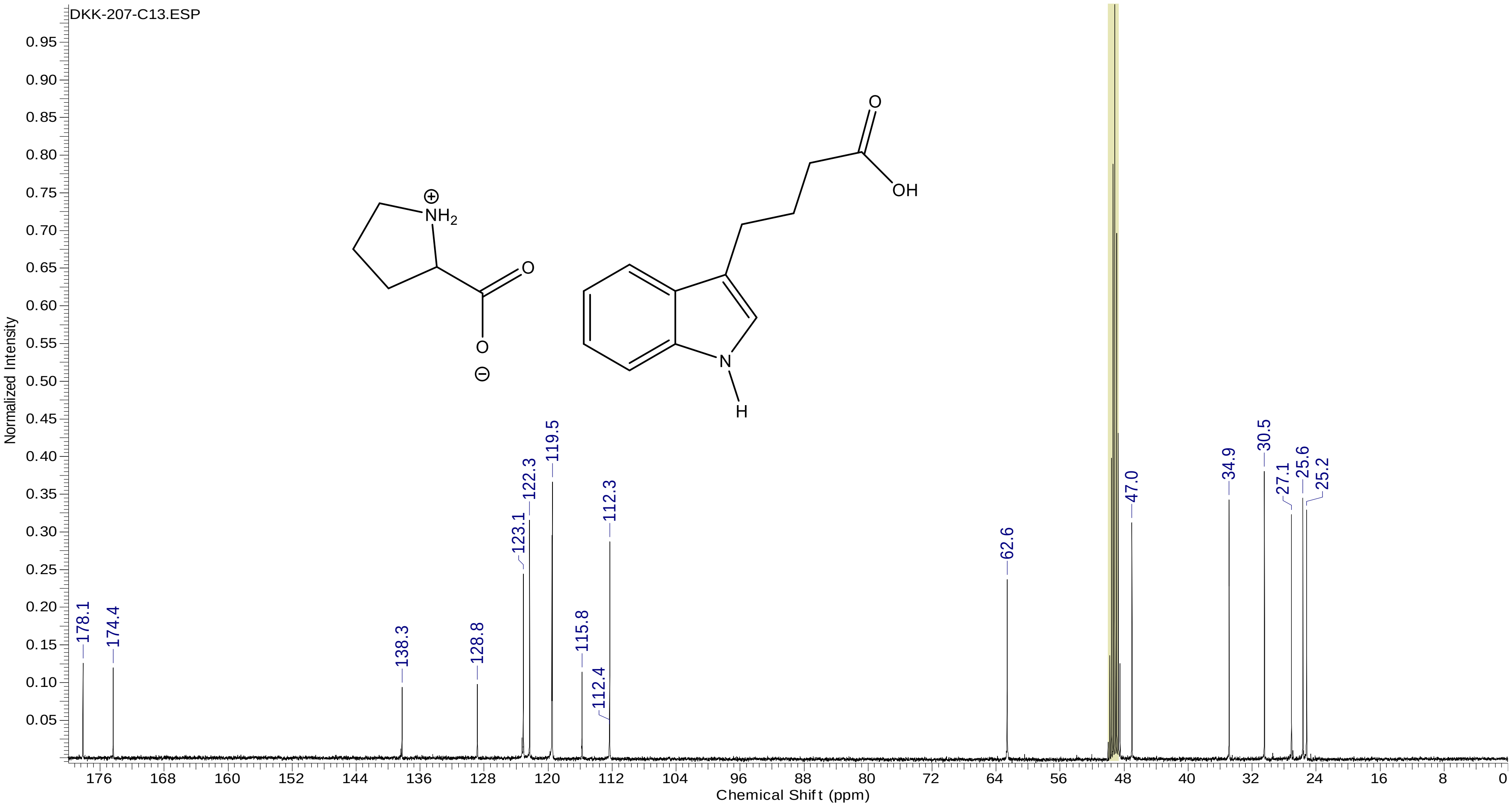

^{13}C NMR (100 MHz, CD_3OD:D_2O 4:1) δ [ppm] = 25.2, 25.6, 27.1, 30.5, 34.9, 47.0, 62.6, 112.3, 112.4, 115.8, 119.5, 122.3, 123.1, 128.8, 138.3, 174.4, 178.1.

Figure S9. UV spectrum of binary mixture of L-proline:indole-3-butyric acid (1:1) (**3**)

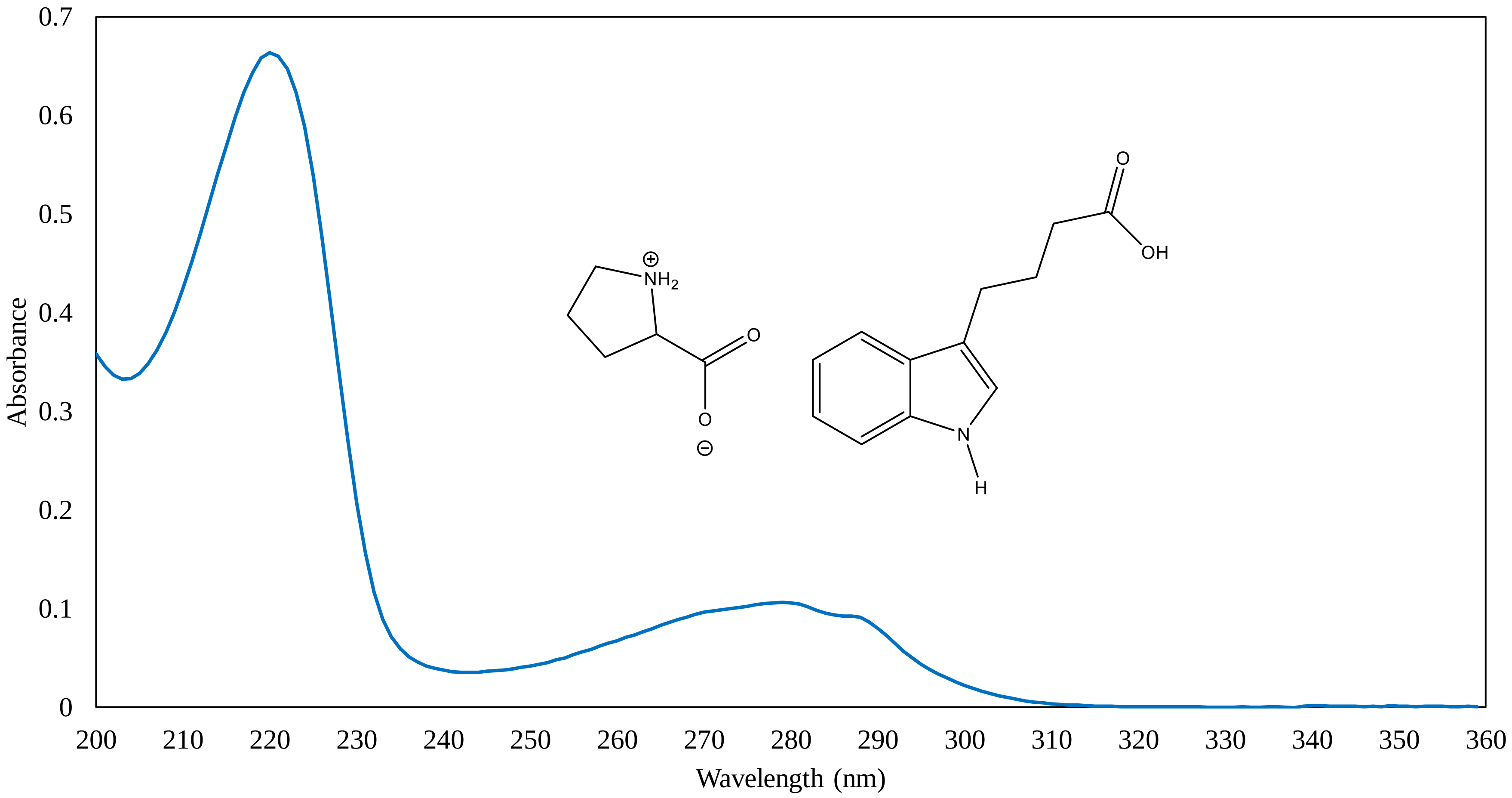

λ_{max} (Water) = 221 nm; $\varepsilon_{\lambda max}$ = 2.69·10^2 dm^3 mol^{-1} cm^{-1}

λ_{max} (Water) = 280 nm; $\varepsilon_{\lambda max}$ = 0.43·10^2 dm^3 mol^{-1} cm^{-1}

Figure S10. ^{1}H NMR spectrum of binary mixture of betaine:indole-3-butyric acid (1:1) (**4**)

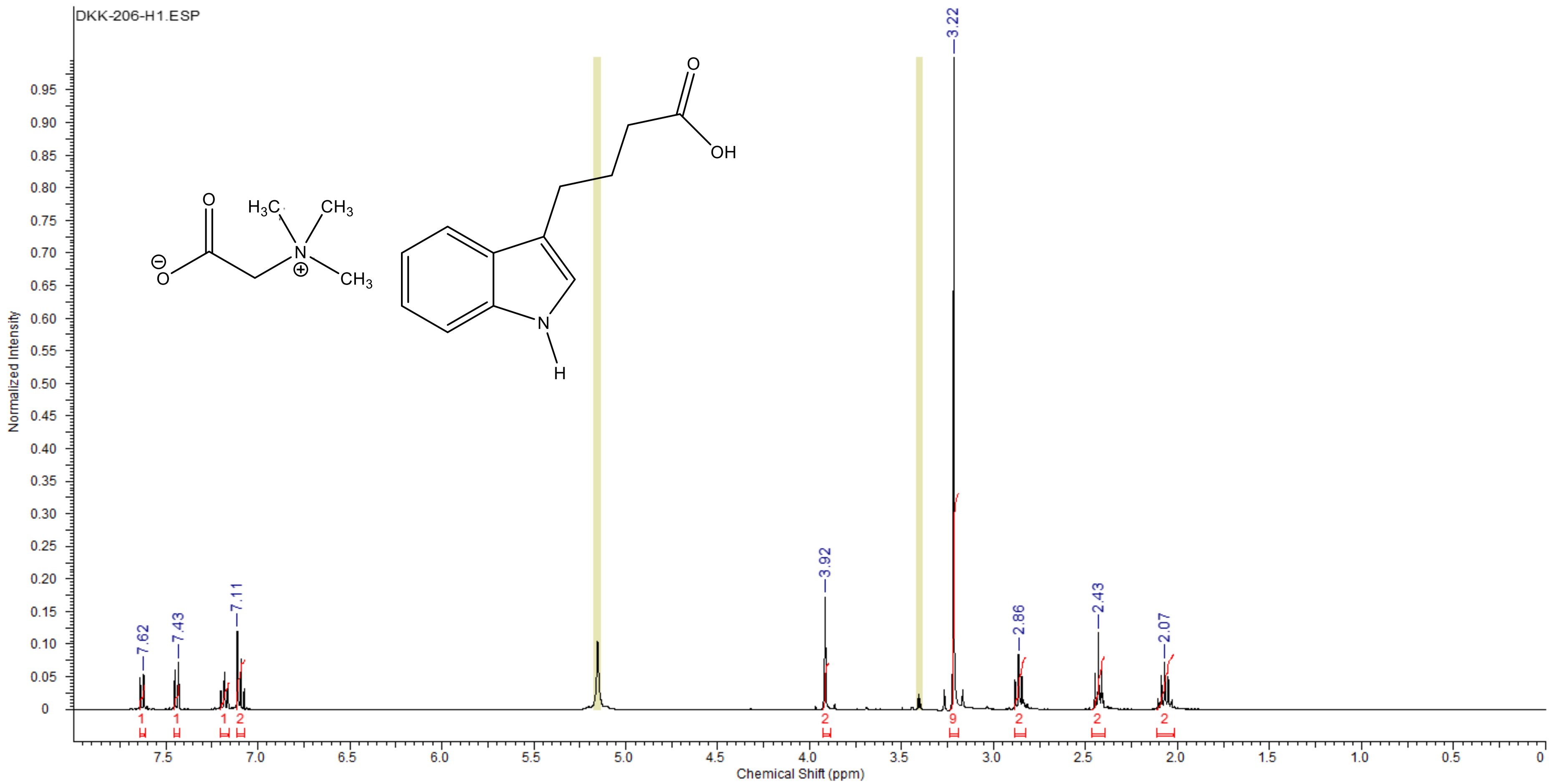

^{1}H NMR (400 MHz, CD_3OD:D_2O 4:1) δ [ppm] = 2.07 (2H, m, $CH_2C\underline{H_2}CH_2COO^-$); 2.43 (2H, m, $CH_2CH_2C\underline{H_2}COO^-$); 2.86 (2H, m, $C\underline{H_2}CH_2CH_2COO^-$); 3.22 (9H, s, $(C\underline{H_3})_3N^+$); 3.92 (2H, s, $N^+C\underline{H_2}COOH$) 7.11 (2H, m, CCHC$\underline{H}$C$\underline{H}$CHC, CC$\underline{H}$NH); 7.43 (1H, m, CCHCHCHC$\underline{H}$C); 7.62 (1H, m, CC$\underline{H}$CHCHCHC).

Figure S11. ^{13}C NMR spectrum of binary mixture of betaine:indole-3-butyric acid (1:1) (**4**)

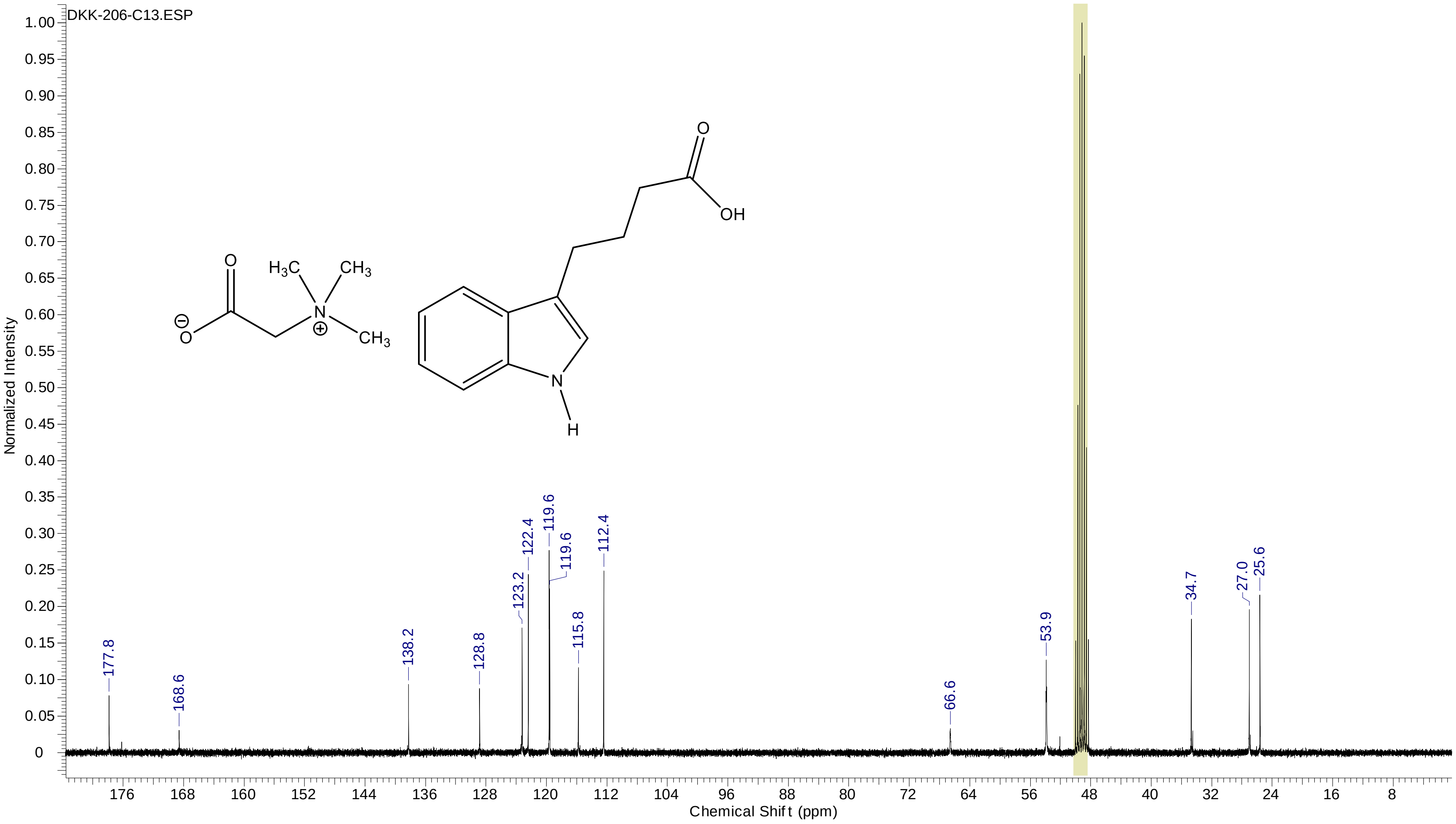

^{13}C NMR (100 MHz, CD_3OD:D_2O 4:1) δ [ppm] = 25.6, 27.0, 34.7, 53.9, 66.6, 112.4, 115.8, 119.6 [2C], 122.4, 123.2, 128.8, 138.8, 168.6, 177.8.

Figure S12. UV spectrum of binary mixture of betaine:indole-3-butyric acid (1:1) (**4**)

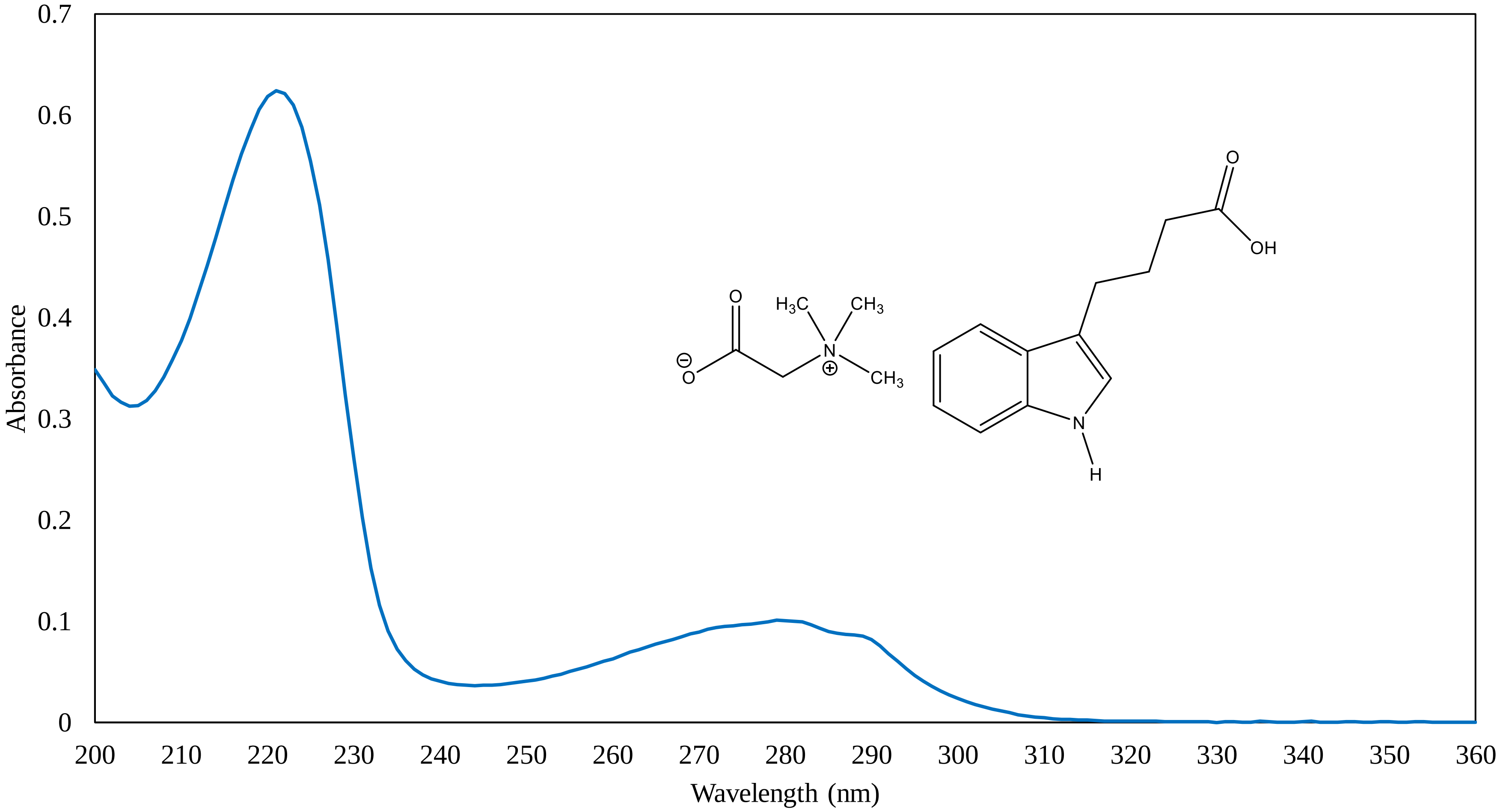

λ_{max} (Water) = 221 nm; $\varepsilon_{\lambda max}$ = $2.52 \cdot 10^2$ dm^3 mol^{-1} cm^{-1}

λ_{max} (Water) = 280 nm; $\varepsilon_{\lambda max}$ = $0.41 \cdot 10^2$ dm^3 mol^{-1} cm^{-1}

Figure S12. ^{1}H NMR spectrum of cholinium indole-3-butyrate (**5**)

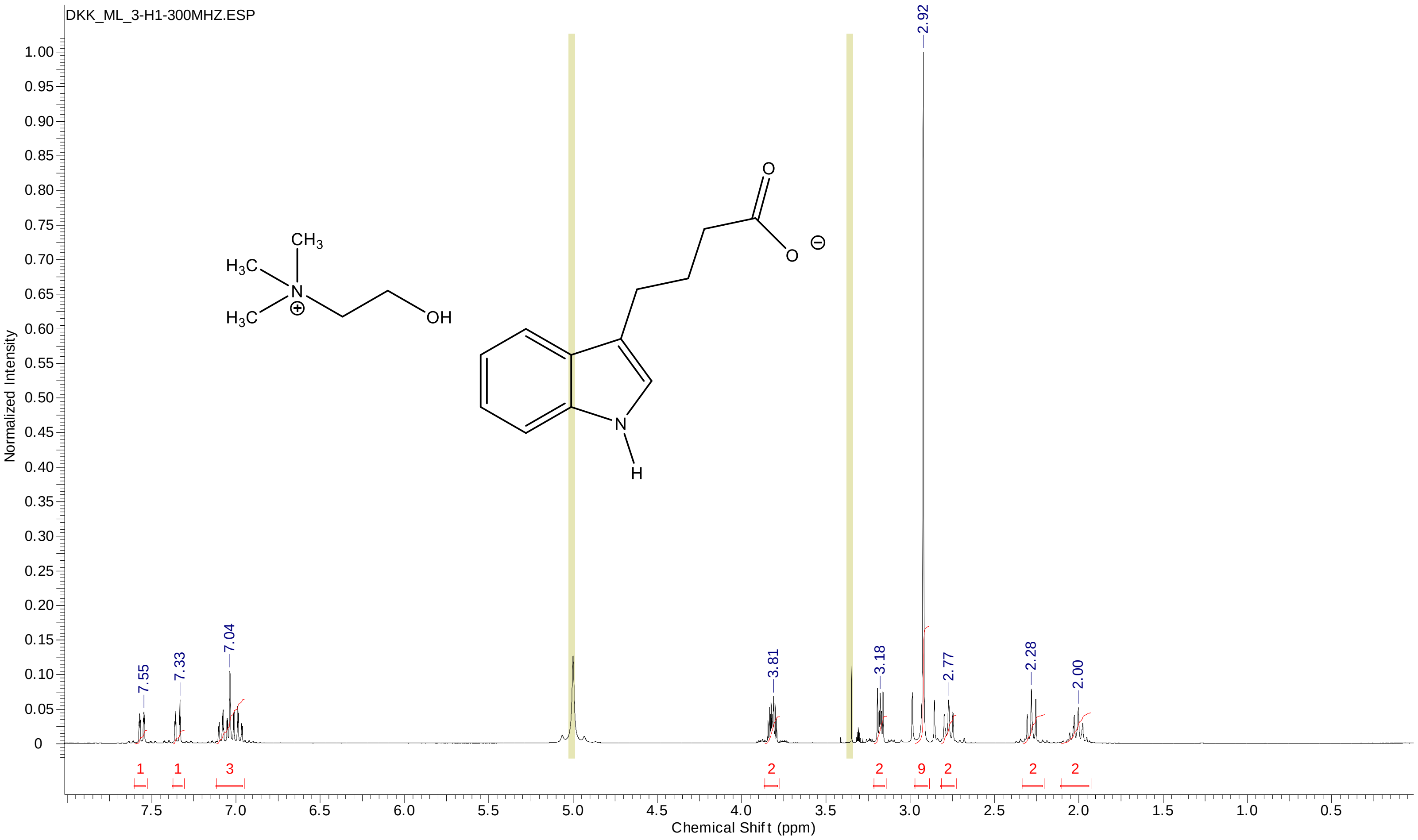

^{1}H NMR (300 MHz, CD_3OD) δ [ppm] = 2.00 (2H, m, -$CH_2C\underline{H}_2CH_2COO^-$); 2.28 (2H, t, *J* = 7.77 Hz, -$CH_2CH_2C\underline{H}_2COO^-$); 2.77 (2H, t, *J* = 7.12 Hz, -$C\underline{H}_2CH_2CH_2COO^-$); 2.92 (9H, s, -$(C\underline{H}_3)_3$); 3.18 (2H, m, $N^+C\underline{H}_2CH_2OH$); 3.81 (2H, m, $N^+CH_2C\underline{H}_2OH$); 7.04 (3H, m, CCHC<u>H</u>C<u>H</u>CHC, NHC<u>H</u>C); 7.33 (1H, d, *J* = 8.02 Hz, CCHCHCHC<u>H</u>C); 7.55 (1H, d, *J* = 7.77 Hz, , CC<u>H</u>CHCHCHC).

Figure S13. ^{13}C NMR spectrum of cholinium indole-3-butyrate (**5**)

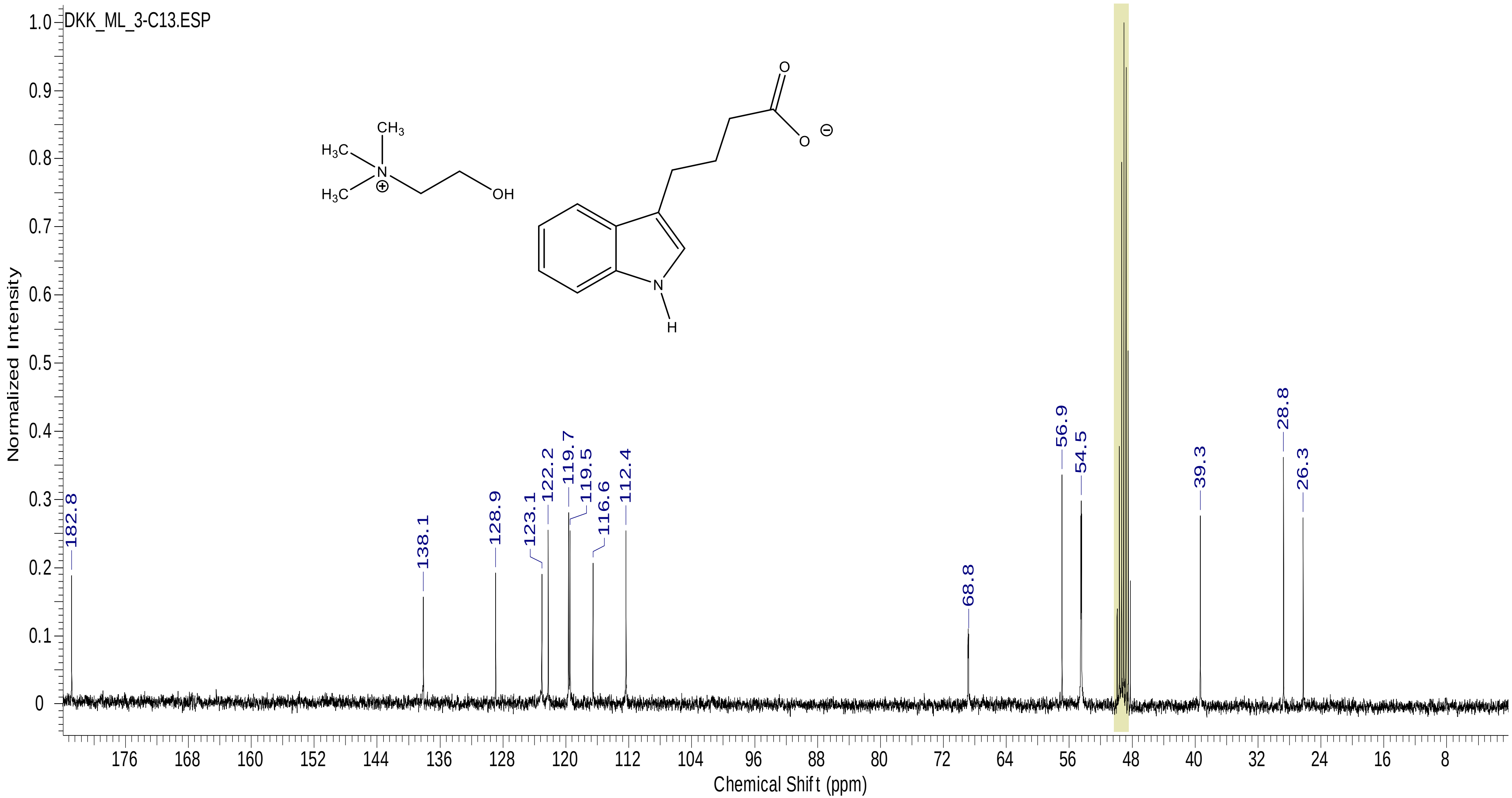

^{13}C NMR (75 MHz, CD_3OD) δ [ppm] = 26.3, 28.8, 39.3, 54.5, 56.9, 68.8, 112.4, 116.6, 119.5, 119.7, 122.2, 123.1, 128.9, 138.1, 182.8.

Figure S14. UV spectrum of cholinium indole-3-butyrate (**5**)

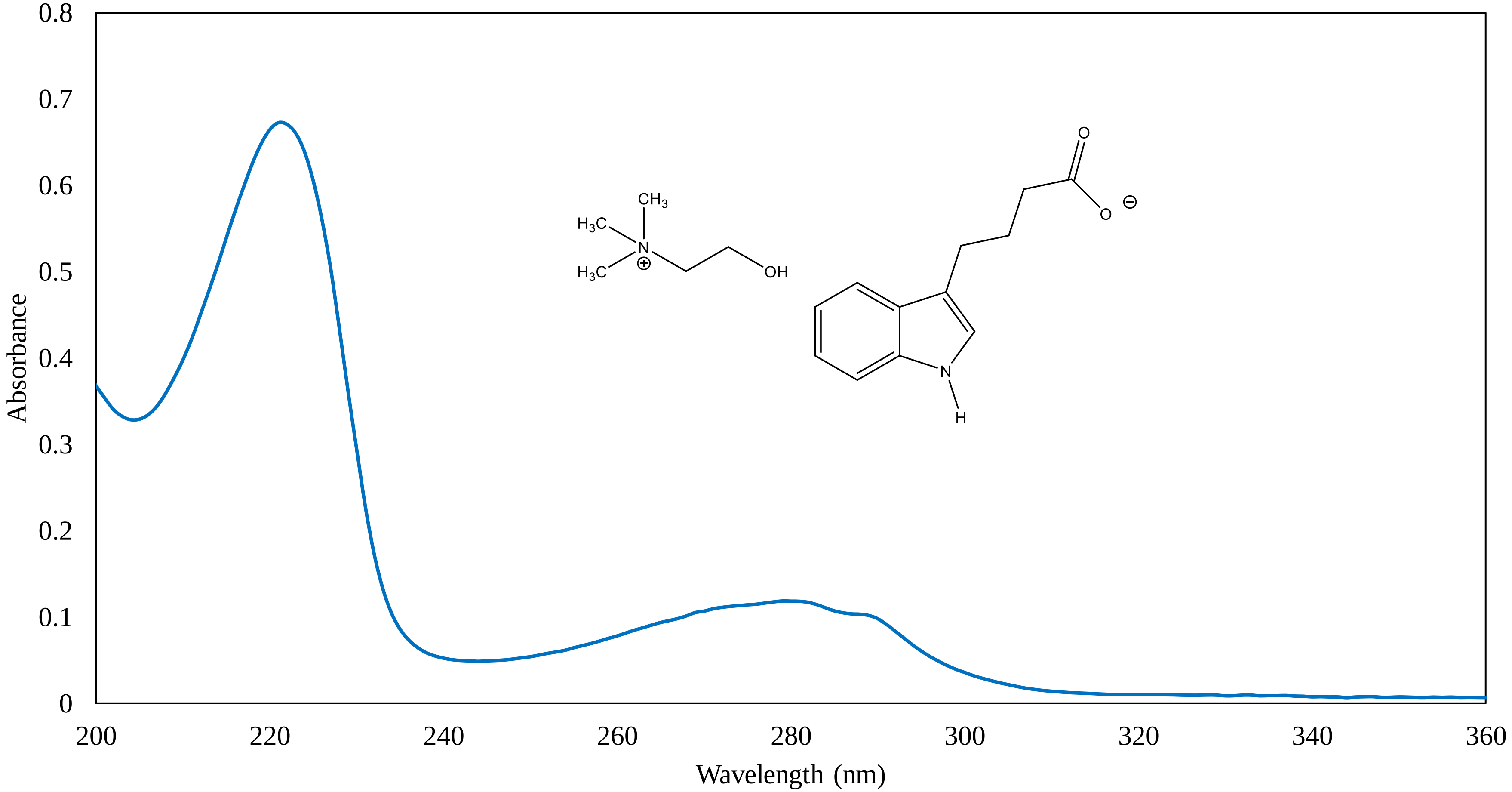

λ_{max} (Water) = 221 nm; $\varepsilon_{\lambda max}$ = 2.72·10^{2} dm^{3} mol^{-1} cm^{-1}

λ_{max} (Water) = 280 nm; $\varepsilon_{\lambda max}$ = 0.48·10^{2} dm^{3} mol^{-1} cm^{-1}

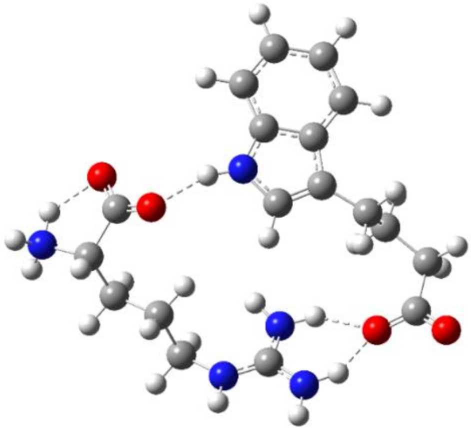

1a

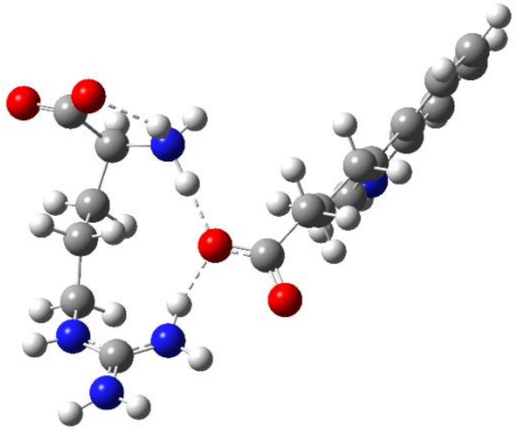

1b

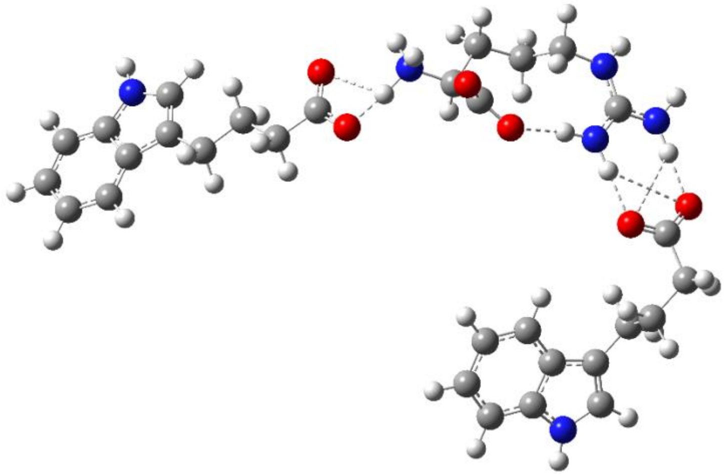

1$_2$

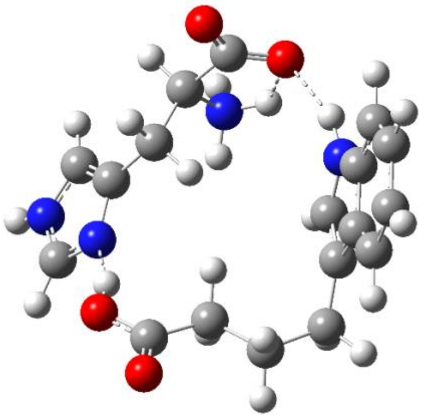

2a

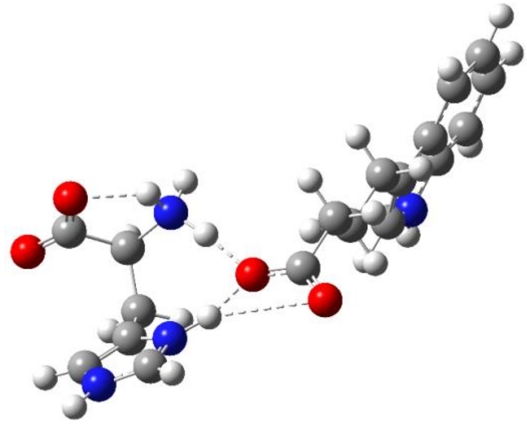

2b

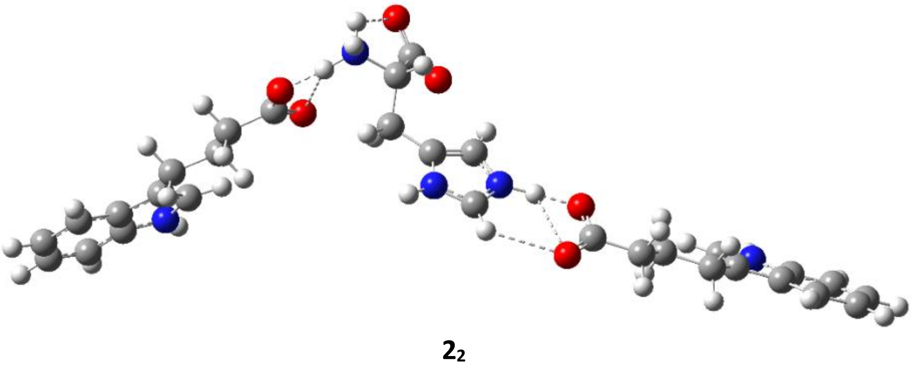

2$_2$

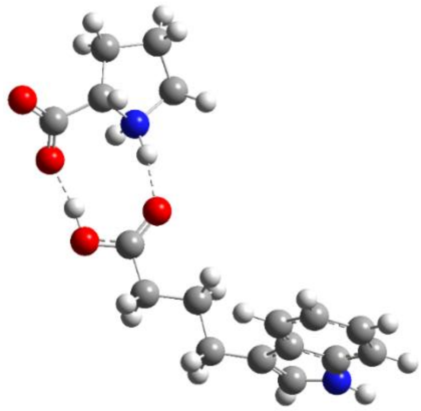

3a

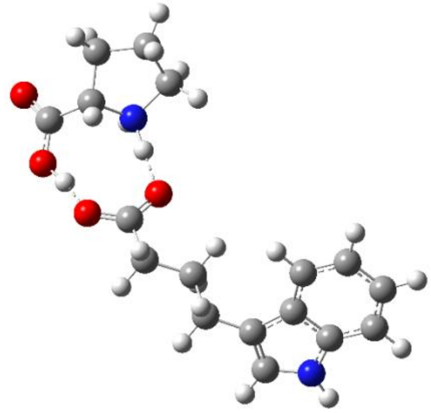

3b

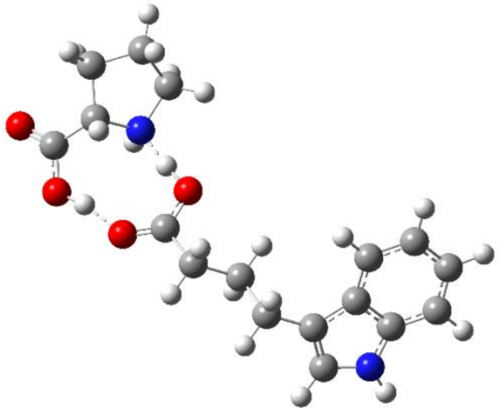

3c

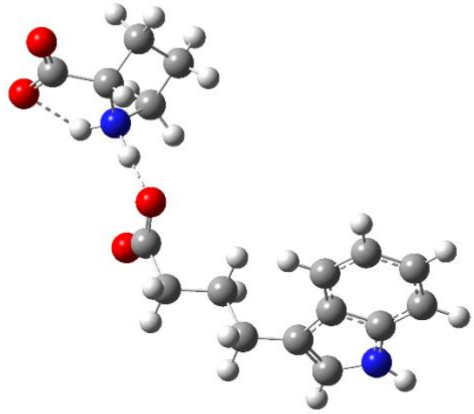

3_1

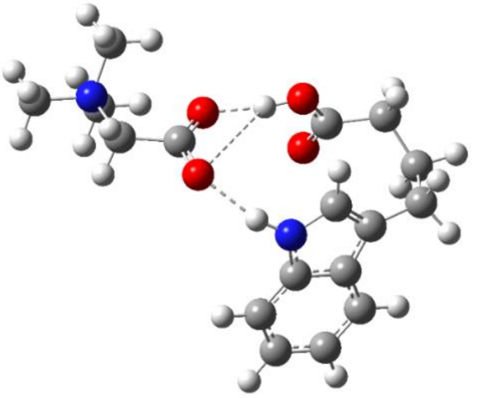

4a

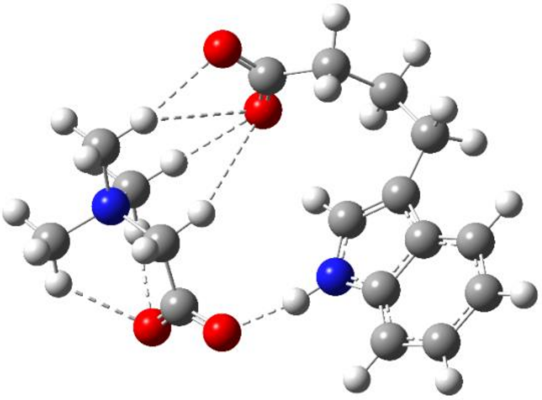

4b

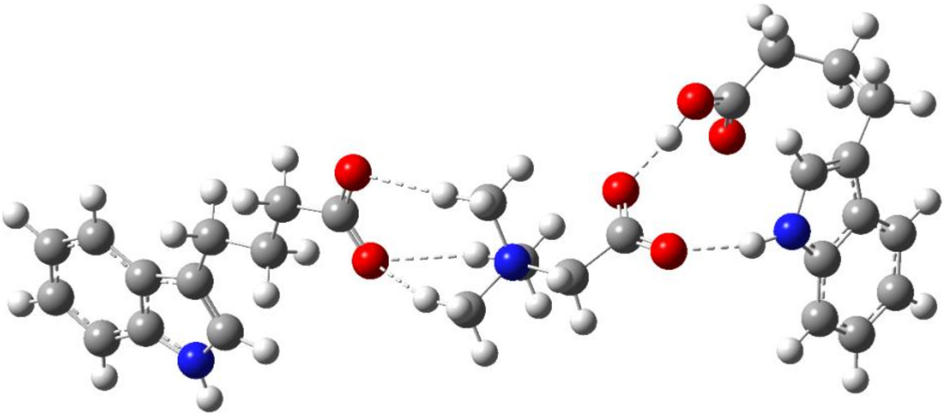

4_2

Figure S15. Possible structures of ammonium salts are shown: **1a, 1b** and **1_2** in a water environment correspond to ionic pairs of a protonated amino acid (charge +1) and an anion of indole-3-butyric acid (charge -1); **2b** and **3b** in water exists as ionic pairs of a protonated amino acid (charge +1) and an anion of indole-3-bututyric acid (charge -1); and binary mixtures **2a**, **3a**, **3c** and **4a** and ionic pairs of glycine betaine and an anion of indole-3-butyric acid **4b** (charge -1). Similarly, 3_1 is an ionic pair of a proline zwitterion and an IBA carboxylate. **2_2** is presented as an ionic pair of protonated amino acids (charge +1) and two anions of ndole-3-butyric acid. The association of glycine betaine and two anions of indole-3-butyric acid **4_2** (total charge -2) is shown

Table S1. Intra- and intermolecular hydrogen bond parameters calculated in water at B3LYP/6-31++G(d,p) level for equimolar binary mixtures and ion pairs of indole-3-butyric acid and arginine (**1_2**), as well as histidine (**2_2**) and betaine (**4_2**)

No	total charge [a.u.]	hydrogen bond	intermolecular hydrogen bond parameters length(Å), angle (degree)
**1_2**	-1	$N^{Arg\_amm}H1 \dots O1^{IBAno1}$	1.509 (173.5)
		$N^{Arg\_amm}H1 \dots O2^{IBAno1}$	2.493 (110.8)
		$N^{Arg\_amm}H \dots O1^{Arg\ a)}$	2.040 (113.5)
		$N^{Arg\_gua}H \dots O2^{Arg\ a)}$	1.875 (171.1)
		$N1^{Arg\_gua}H \dots O1^{IBAno2}$	1.756 (179.5)
		$N1^{Arg\_gua}H \dots O2^{IBAno2}$	2.882 (128.5)
		$N2^{Arg\_gua}H \dots O2^{Bano2}$	1.723 (179.1)
		$N2^{Arg\_gua}H \dots O1^{IBAno2}$	2.829 (128.0)
**2_2**	-1	$N^{His\_amm}H \dots O1^{IBAno1}$	1.490 (172.5)
		$N^{His\_amm}H \dots O2^{IBAno1}$	2.701 (131.1)
		$N^{His\_amm}H \dots O1^{His\ a)}$	1.962 (117.4)
		$N1^{His\_Im}H \dots O1^{IBAno2}$	1.449 (178.1)
		$N1^{His\_Im}H \dots O2^{IBAno2}$	2.538 (116.5)
		$C2^{His\_Im}H \dots O2^{IBAno2}$	2.565 (115.7)
**4_2**	-1	$C^{Met1}H \dots O1^{IBAno1}$	2.290 (174.1)
		$C^{Met1}H \dots O2^{IBAno1}$	2.917 (136.3)
		$C^{Met2}H \dots O2^{IBAno1}$	2.299 (156.0)
		$C^{Met3}H \dots O2^{IBAno1}$	2.382 (153.1)
		$C^{Met1}H \dots O1^{Bet\ b)}$	2.336 (121.9)
		$C^{Met3}H \dots O1^{Bet\ b)}$	2.313 (121.7)
		$C(O)O1H^{IBAno2} \dots O1^{Bet}$	1.617 (170.0)
		$C(O)O1H^{IBA} \dots O2^{Bet}$	3.070 (132.9)
		$N^{IBA\_Ind}H \dots O2^{Bet}$	1.903 (162.1)

a) intramolecular hydrogen bond between ammonium group and carboxylate of arginine;
b) intramolecular hydrogen bond between N-methyl group and carboxylate of betaine;
c) binary mixture.

Table S2. Complexation energy of ionic or neutral components of ion pairs or binary mixtures $\mathbf{1_2}$, $\mathbf{2_2}$, and $\mathbf{4_2}$ in a water environment for geometries calculated at B3LYP/6-31++G(d,p) level

No	total charge [a.u.]	class of junction	complexation energy [kcal/mole]
$\mathbf{1_2}$	-1	amonium ($^{+}NH_3$ + acid anion)	-17.1
		guanidinium ($^{+}$gua + acid anion)	-16.7
$\mathbf{2_2}$	-1	amonium ($^{+}NH_3$ + acid anion)	-19.9
		imidazolium ($^{+}$his + acid anion)	-8.9
$\mathbf{4_2}$	-1	carboxylate (carboxylate anion of betaine + acid)	-14.7
		tetraalkiloammonium ($^{+}N(CH_3)_3$ + acid anion)	-2.7

Table S3. Electronic energy (Hartree) of ion pairs or binary mixtures of arginine, histidine and betaine with acidic (anionic) IBA in a water environment for geometries calculated at B3LYP/6-31++G(d,p) level for a 1:2 molar ratio

No	total charge [a.u.]	class of compound	type of interaction	[Hartree]
$\mathbf{1_2}$	-1	protonated aminoacid + two anion	$^{+}$gua - $^{-}O_2C$<br>$^{+}NH_3$ - $^{-}O_2C$	-1277.0433542
$\mathbf{2_2}$	-1	protonated aminoacid + two anion	$^{+}$imid - $^{-}O_2C$<br>$^{+}NH_3$ - $^{-}O_2C$	-1889.1836824
$\mathbf{4_2}$	-1	betaine + acid anion + acid	C(O)OH - $^{-}O_2C$<br>$^{+}NH_3$ - $^{-}O_2C$	-1072.8022953

Figure S16. ^{13}C NMR spectrum of L-arginine, L-arginine HCl, and **1**

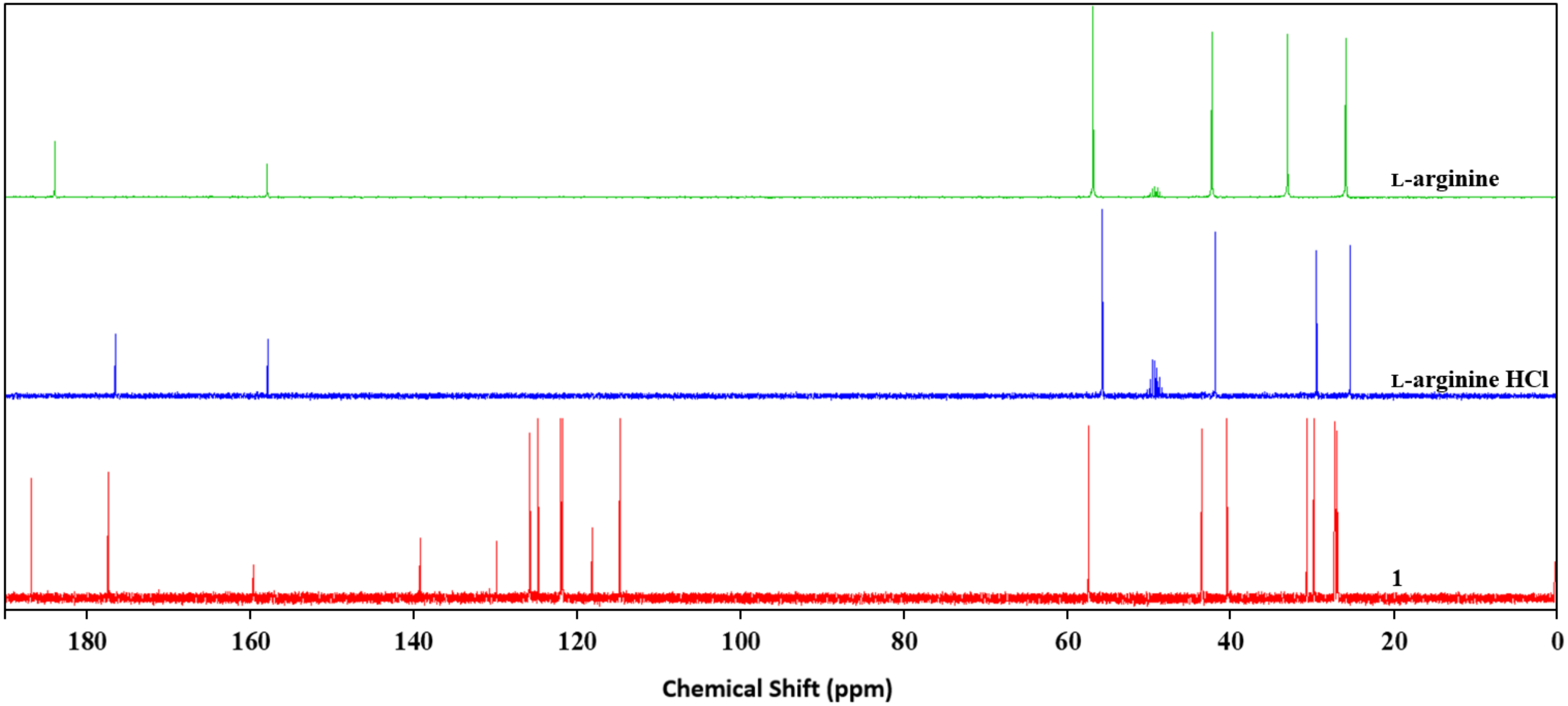

Figure S17. ^{1}H NMR spectrum of L-histidine, IBA, L-histidine HCl, and **2**

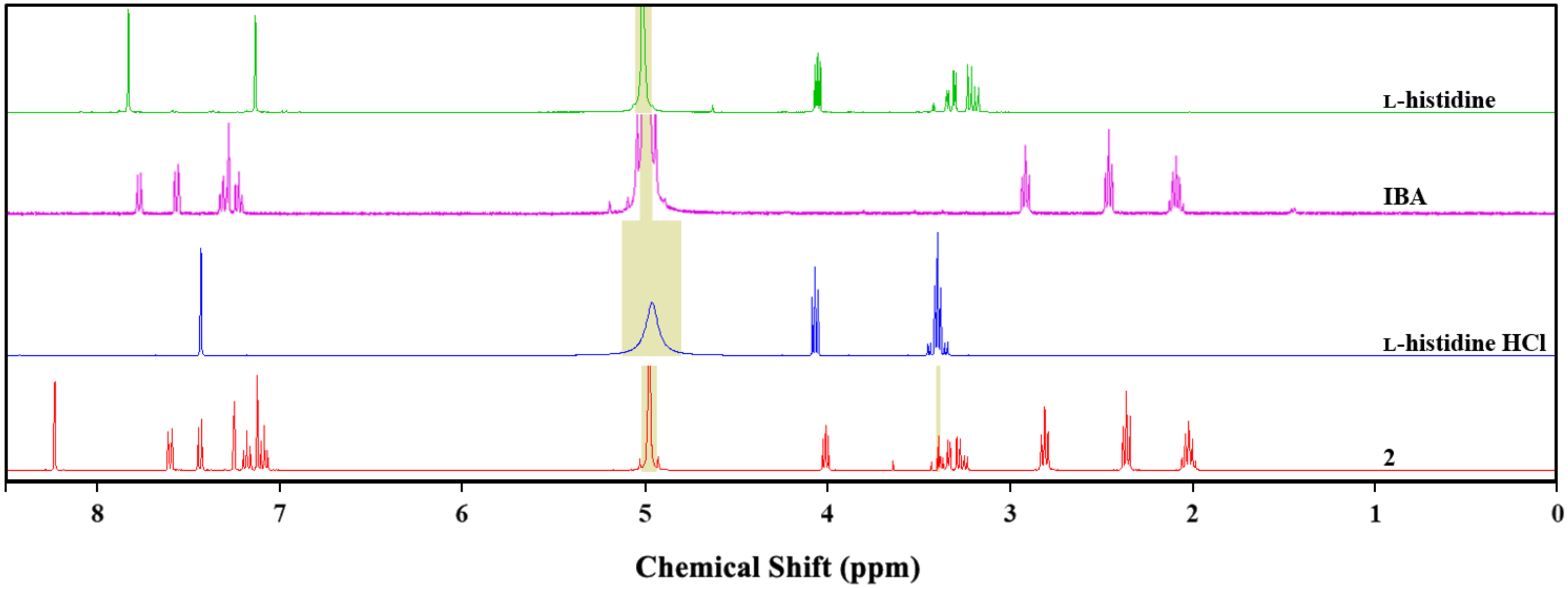

Figure S18. ^{13}C NMR spectrum of L-histidine, L-histidine HCl, and **2**

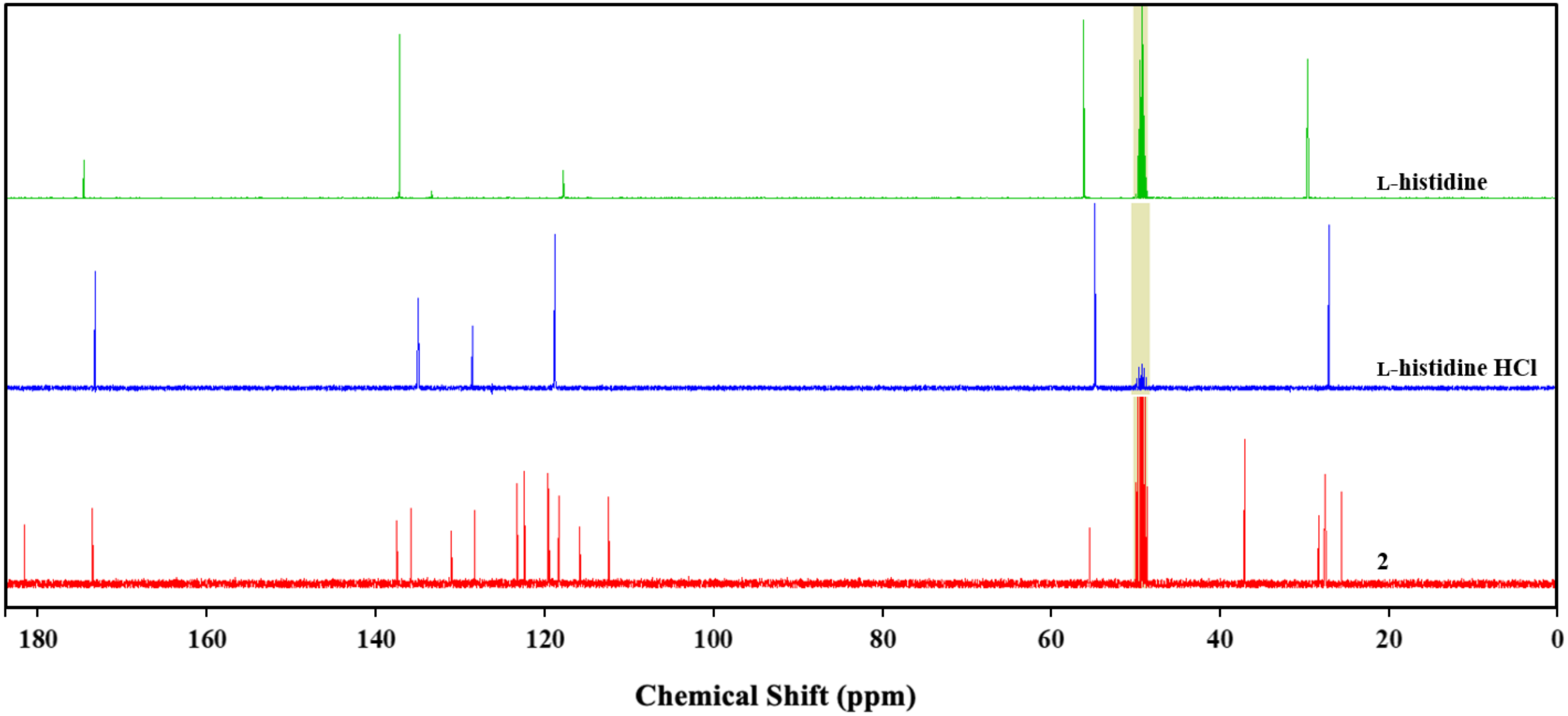

Figure S19. ^{13}C NMR spectrum of L-proline, L-proline HCl, and **3**

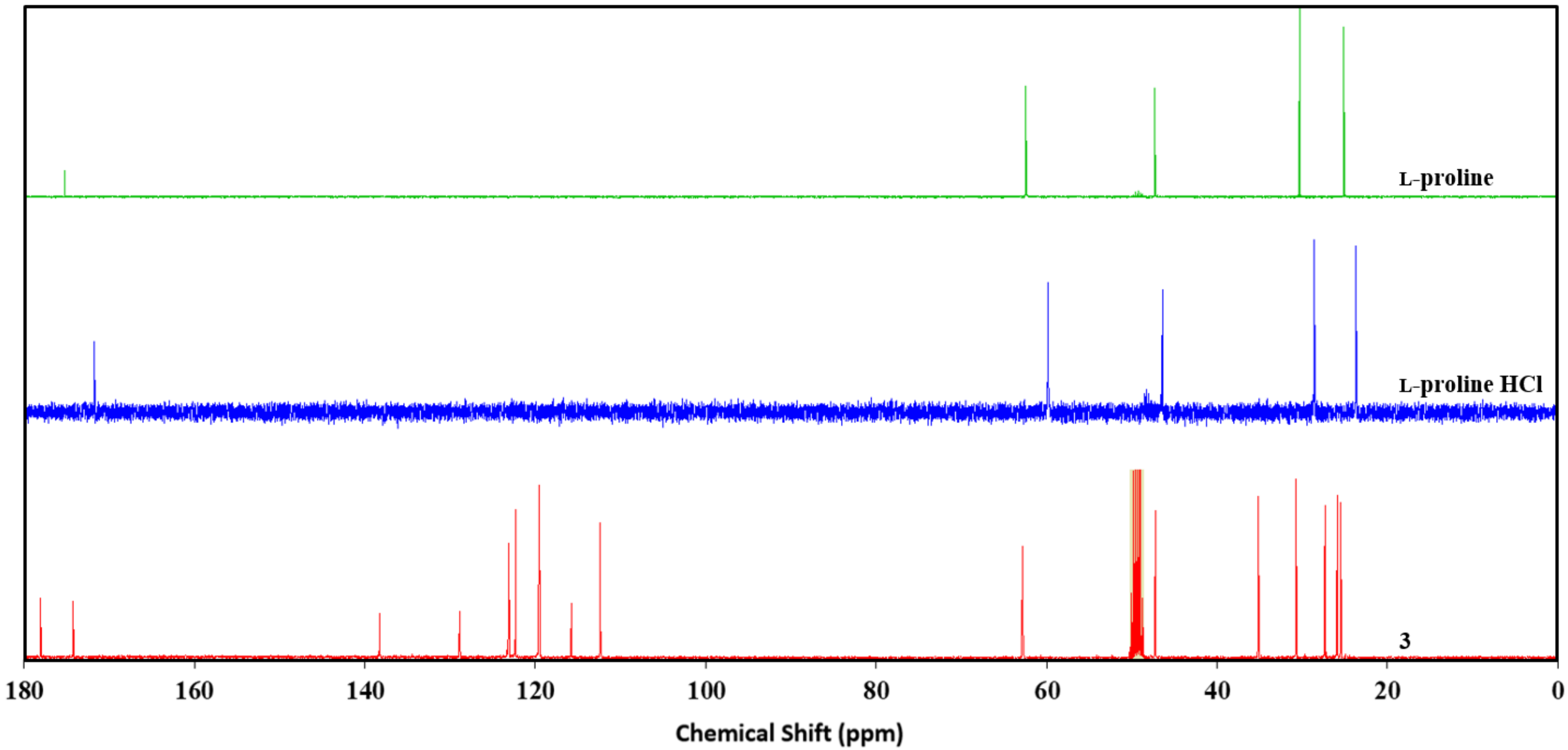

Figure S20. ^{1}H NMR spectrum of betaine, IBA, betaine HCl, and **4**

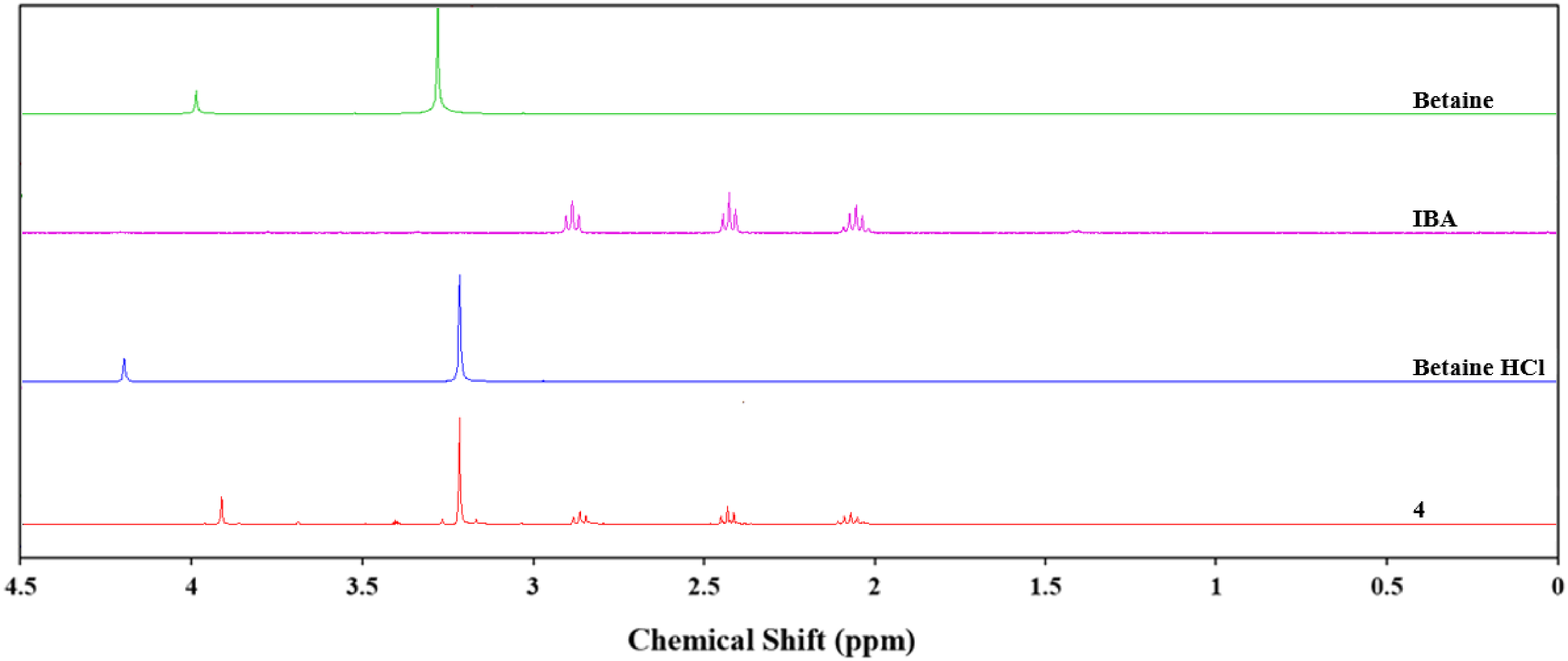

Figure S21. ^{13}C NMR spectrum of betaine, betaine HCl, and **4**

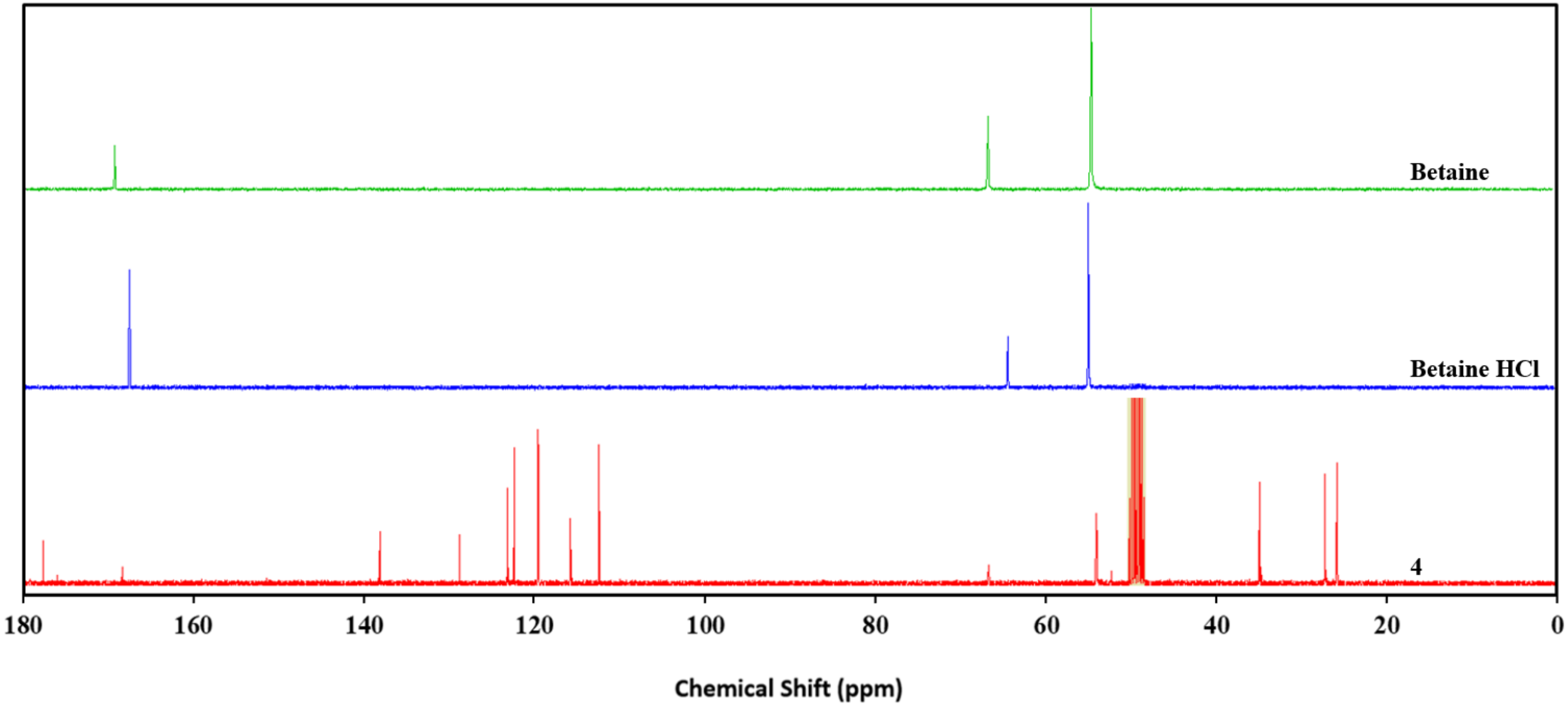

Table S4. Solubility of prepared products at 25 °C

No	Water	Methanol	DMSO	Acetonitrile	Acetone	Isopropanol	Chloroform
**1**	±	±	–	–	–	–	–
**2**	±	–	–	–	–	–	–
**3**	±	+	–	–	+	–	–
**4**	±	+	–	–	+	–	–
**5**[a]	+	+	–	–	+	–	–
**IBA**	–	+	+	±	±	–	+

+: good solubility; ±: limited solubility; –: poor solubility; [a]Data from literature[1]

Table S5. Changes in IBA content for **1**, **2**, **3**, **4**, **5** and indole-3-butyric acid (**IBA**) during storage in water in the dark

Time [Days]	Content of IBA [%]					
	**IBA**	**1**	**2**	**3**	**4**	**5**
0	100.00±0.2	100.00±0.5	100.00±0.8	100.00±0.3	100.00±0.6	100.00±0.3
1	99.98±1.0	99.96±0.3	99.35±0.3	99.98±0.4	99.76±0.4	99.97±0.4
7	99.88±0.2	99.95±0.8	99.30±0.7	99.82±0.9	99.71±0.6	99.78±0.1
14	99.81±0.3	99.93±0.6	98.97±0.9	99.78±0.6	99.63±0.1	99.74±0.6
28	99.75±0.5	99.62±0.4	98.44±0.5	99.66±0.1	99.60±0.2	99.71±0.3

Table S6. Degradation of IBA in **1**, **2**, **3**, **4**, **5** and indole-3-butyric acid (**IBA**) during storage in water in the dark

Time [Days]	**IBA**	**1**	**2**	**3**	**4**	**5**
0	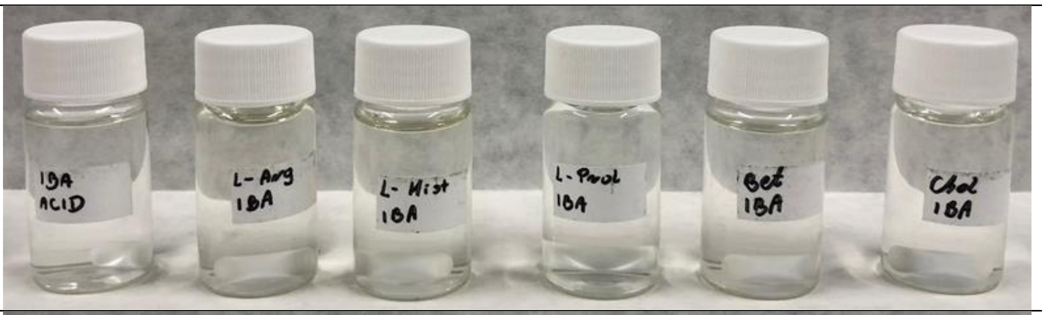					
1	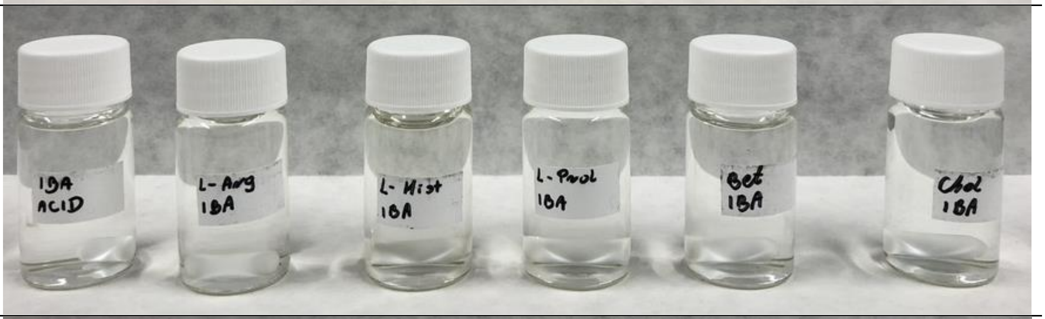					
7	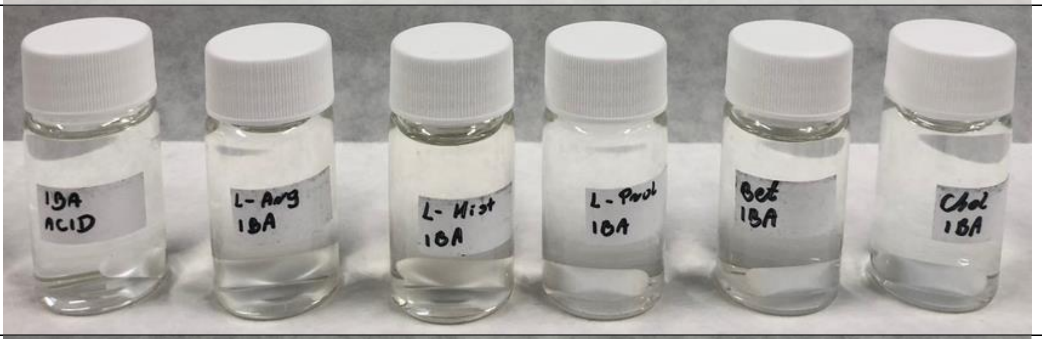					
14	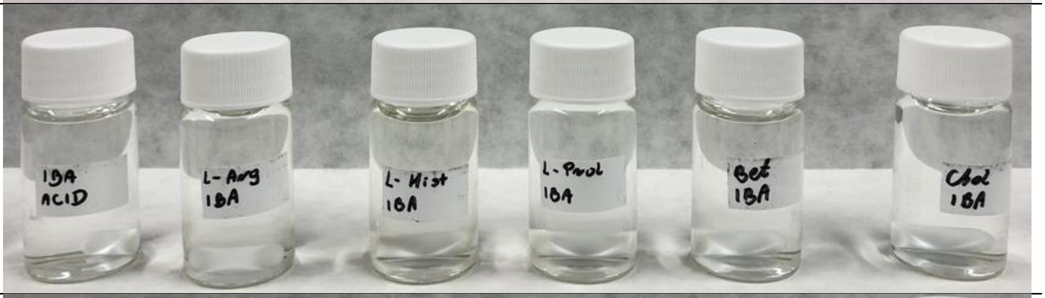					
28	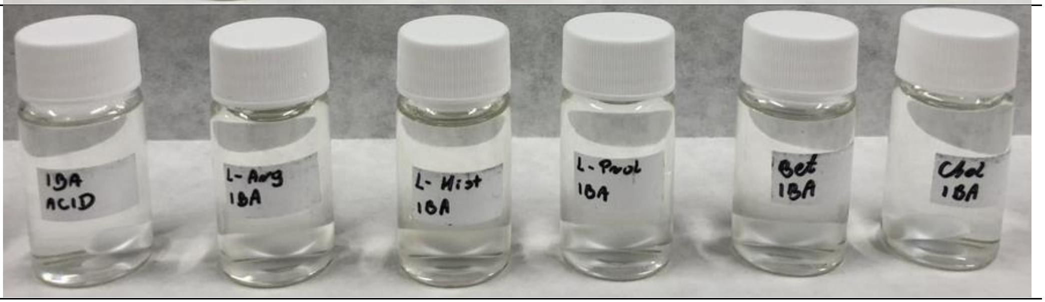					

Table S7. Changes in IBA content for **1**, **2**, **3**, **4**, **5** and indole-3-butyric acid (**IBA**) during storage in water in the light.

Time [Days]	Content of IBA [%]					
	**IBA**	**1**	**2**	**3**	**4**	**5**
0	100.00±0.2	100.00±0.5	100.00±0.7	100.00±1.3	100.00±0.5	100.00±0.8
1	99.85±1.1	99.90±0.9	99.93±0.4	99.71±0.6	99.92±0.1	99.57±0.6
7	99.46±0.5	99.60±1.1	98.68±0.9	99.27±0.4	99.56±0.6	99.49±0.1
14	99.17±0.6	96.23±0.3	88.96±0.6	98.84±0.8	98.49±0.1	93.72±0.6
28	93.40±0.1	82.21±0.4	64.19±1.2	84.08±0.7	88.22±0.2	80.61±0.5

Table S8. Degradation of IBA in **1**, **2**, **3**, **4**, **5** and indole-3-butyric acid (**IBA**) during storage in water in the light

Time [Days]	**IBA**	**1**	**2**	**3**	**4**	**5**
0	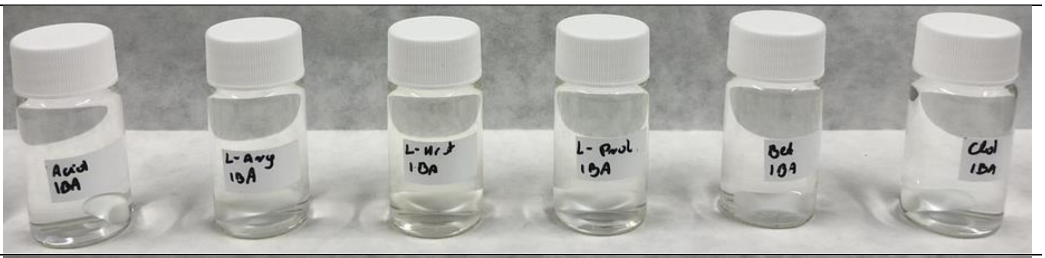					
1	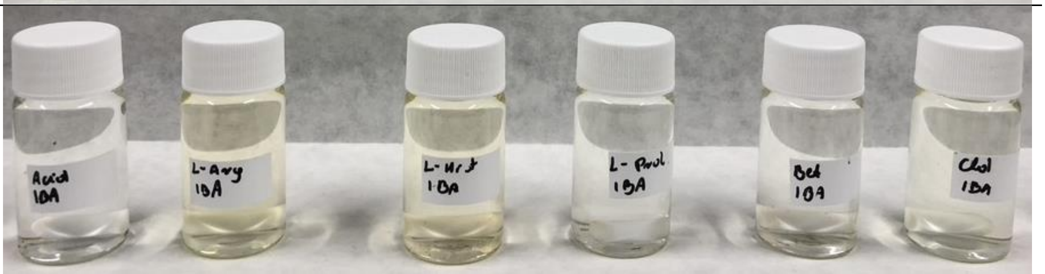					
7	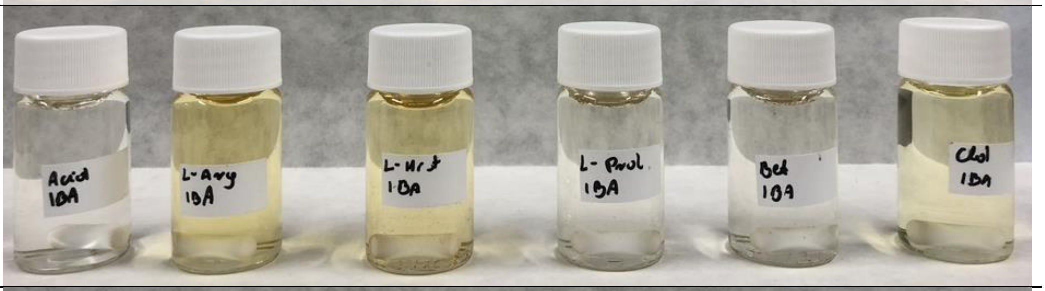					
14	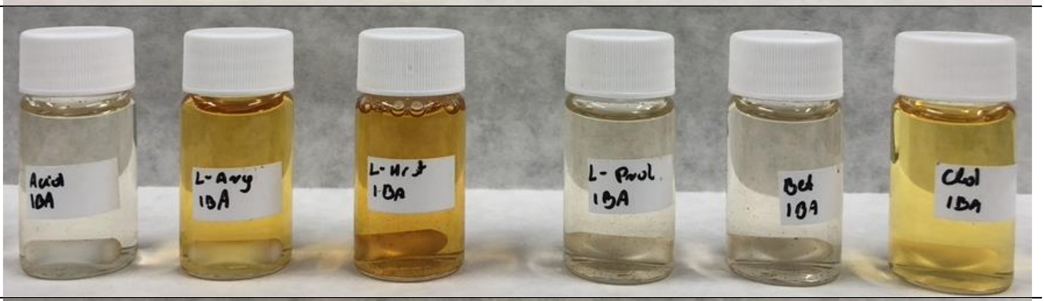					
28	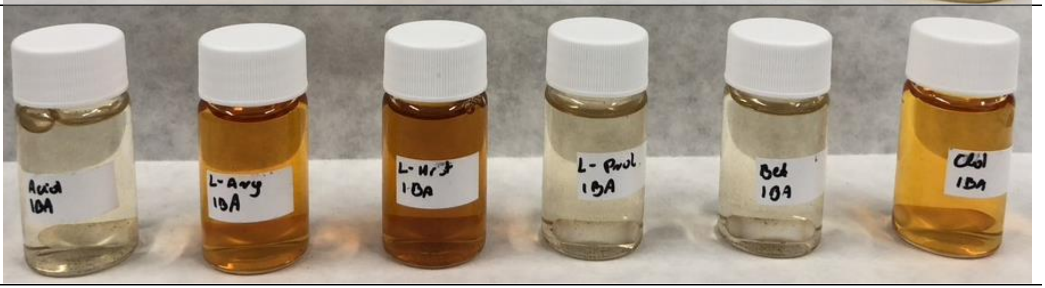					

Table S9. Influence of ammonium salts and binary mixtures containing indole-3-butric acid on seed mustard germination capacity

**No**	**concentration [mg L^{-1}]**	**I day [%] ± St. Dev.**	**II day [%] ± St. Dev.**	**III day [%] ± St. Dev.**	**IV day [%] ± St. Dev.**	**V day [%] ± St. Dev.**	**VI day [%] ± St. Dev.**	**VII day [%] ± St. Dev.**
**1**	25	0.00 ± 0.0	86.67 ± 0.58	86.67 ± 0.58	86.67 ± 0.58	86.67 ± 0.58	86.67 ± 0.58	86.67 ± 0.58
	50	6.67 ± 0.58	73.33 ± 0.58	80.00 ± 0.58	80.00 ± 0.58	80.00 ± 0.58	86.67 ± 0.58	86.67 ± 0.58
	100	6.67 ± 0.58	86.67 ± 0.58	86.67 ± 0.58	86.67 ± 0.58	86.67 ± 0.58	86.67 ± 0.58	86.67 ± 0.58
**2**	25	10.00 ± 1.73	80.00 ± 0.0	90.00 ± 1.0	90.00 ± 1.0	90.00 ± 1.0	90.00 ± 1.0	93.33 ± 1.0
	50	20.00 ± 1.00	80.00 ± 0.0	80.00 ± 1.0	80.00 ± 1.0	80.00 ± 1.0	86.67 ± 1.0	86.67 ± 1.0
	100	6.67 ± 0.58	20.00 ± 1.00	40.00 ± 1.0	40.00 ± 1.0	40.00 ± 1.0	40.00 ± 1.0	40.00 ± 1.0
**3**	25	3.33 ± 0.58	66.67 ± 0.58	73.33 ± 0.58	73.33 ± 0.58	73.33 ± 0.58	76.67 ± 0.58	76.67 ± 0.58
	50	6.67 ± 1.15	73.33 ± 0.58	93.33 ± 0.58	93.33 ± 0.58	93.33 ± 0.58	96.67 ± 0.58	96.67 ± 0.58
	100	0.00 ± 0.0	13.33 ± 1.15	13.33 ± 1.15	13.33 ± 1.15	13.33 ± 1.15	13.33 ± 1.15	13.33 ± 1.15
**4**	25	0.00 ± 0.0	73.33 ± 1.53	83.33 ± 0.58	83.33 ± 0.58	83.33 ± 0.58	86.67 ± 0.58	86.67 ± 0.58
	50	0.00 ± 0.0	26.67 ± 1.15	60.00 ± 0.0	60.00 ± 0.0	60.00 ± 0.0	76.67 ± 0.0	76.67 ± 0.0
	100	6.67 ± 1.15	33.33 ± 1.53	63.33 ± 2.08	63.33 ± 2.08	63.33 ± 2.08	63.33 ± 2.08	73.33 ± 2.08
**5**	25	26.67 ± 0.58	56.67 ± 1.53	66.67 ± 0.58	66.67 ± 0.58	66.67 ± 0.58	66.67 ± 0.58	66.67 ± 0.58
	50	16.67 ± 0.58	66.67 ± 0.58	86.67 ± 0.58	86.67 ± 0.58	86.67 ± 0.58	86.67 ± 0.58	86.67 ± 0.58
	100	10.00 ± 0.0	53.33 ± 2.52	86.67 ± 0.58	86.67 ± 0.58	86.67 ± 0.58	86.67 ± 0.58	86.67 ± 0.58
**REF**	25	0.00 ± 0.0	30.00 ± 0.0	43.33 ± 0.58	43.33 ± 1.0	43.33 ± 0.58	56.67 ± 0.58	50.00 ± 0.58
	50	0.00 ± 0.0	10.00 ± 1.0	50.00 ± 1.0	50.00 ± 1.53	50.00 ± 1.0	50.00 ± 1.0	60.00 ± 1.0
	100	0.00 ± 0.0	10.00 ± 1.0	30.00 ± 1.0	30.00 ± 0.58	30.00 ± 1.0	33.33 ± 1.0	33.33 ± 1.0

Figure S22. Effect of ammonium salts and binary mixtures containing indole-3-butric acid at 50 mg L^{-1} concentration on mustard shoot and root lengths

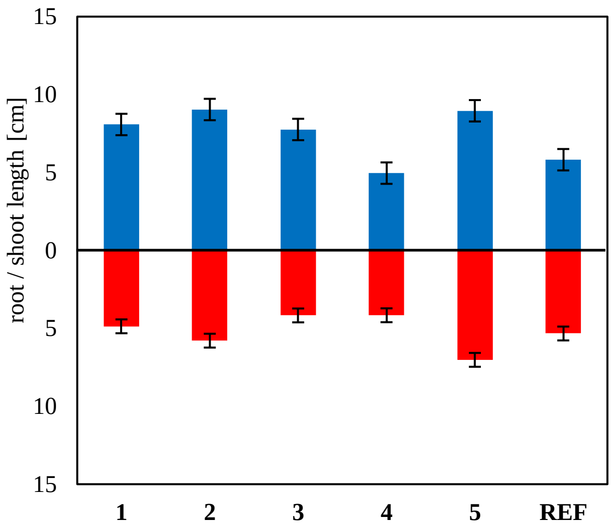

Figure S23. Effect of ammonium salts and binary mixtures containing indole-3-butric acid at 100 mg L^{-1} concentration on mustard shoot and root lengths

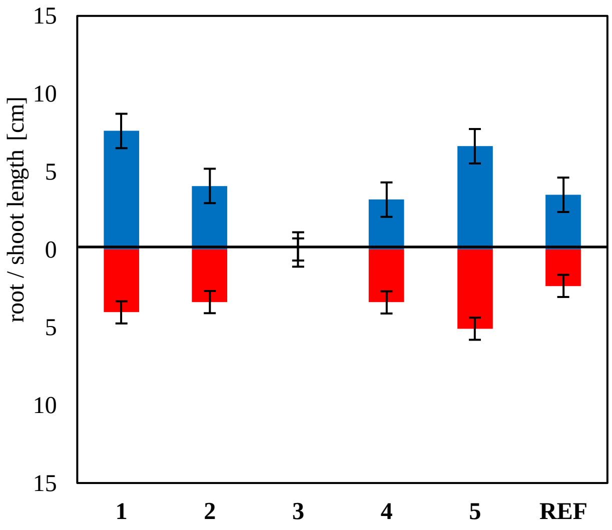

REFERENCES

1. D.K. Kaczmarek, T. Kleiber, L. Wenping, M. Niemczak, L. Chrzanowski, J. Pernak, *ACS Sustainable Chem. Eng.*, 2020, **8**, 1591–1598.
2. M. Niemczak, D.K. Kaczmarek, T. Klejdysz, D. Gwiazdowska, K. Marchwińska, J. Pernak, *ACS Sustainable Chem. Eng.*, 2019, **7**, 1072–1084.
3. A.I. Vogel, B.S. Furniss, A.J. Hannaford, V. Rogers, P.W.G. Smith, A.R. Tatchell, *Vogel`s Textbook of Practical Organic Chemistry*, 5th ed., Longman Scientific & Technical, Harlow, UK, 1989.
4. A.D. Becke, *J. Chem. Phys.*, 1993, **98**, 5648–5652.
5. P.A. Hunt, B. Kirchner, T. Welton, *Chem. – Eur. J.*, 2006, **12**, 6762–6775.
6. M. Lozynski, J. Pernak, Z. Gdaniec, B. Gorska, F. Béguin, *Phys. Chem. Chem. Phys.*, 2017, **19**, 25033–25043.
7. K. Dong, S. Zhang, D. Wang, X. Yao, *J. Phys. Chem. A*, 2006, **110**, 9775–9782.
8. K. Fumino, A. Wulf, R. Ludwig, *Phys. Chem. Chem. Phys.*, 2009, **11**, 8790–8794.
9. M. Shyama, S. Lakshmipathi, *J. Mol. Liq.*, 2020, **304**, 112720.
10. M. J. Frisch, et al., *Gaussian 09*, revision B.01, Gaussian, Inc., Wallingford, CT, 2010.
